# The Importance of Childhood for Adult Health and Development—Study Protocol of the Zurich Longitudinal Studies

**DOI:** 10.3389/fnhum.2020.612453

**Published:** 2021-01-28

**Authors:** Flavia M. Wehrle, Jon Caflisch, Dominique A. Eichelberger, Giulia Haller, Beatrice Latal, Remo H. Largo, Tanja H. Kakebeeke, Oskar G. Jenni

**Affiliations:** ^1^Child Development Center, University Children's Hospital Zurich, Zurich, Switzerland; ^2^Children's Research Center, University Children's Hospital Zurich, Zurich, Switzerland; ^3^University of Zurich, Zurich, Switzerland

**Keywords:** longitudinal study, study protocol, development, growth, childhood, lifespan, aging

## Abstract

Evidence is accumulating that individual and environmental factors in childhood and adolescence should be considered when investigating adult health and aging-related processes. The data required for this is gathered by comprehensive long-term longitudinal studies. This article describes the protocol of the Zurich Longitudinal Studies (ZLS), a set of three comprehensive cohort studies on child growth, health, and development that are currently expanding into adulthood. Between 1954 and 1961, 445 healthy infants were enrolled in the first ZLS cohort. Their physical, motor, cognitive, and social development and their environment were assessed comprehensively across childhood, adolescence, and into young adulthood. In the 1970s, two further cohorts were added to the ZLS and assessed with largely matched study protocols: Between 1974 and 1979, the second ZLS cohort included 265 infants (103 term-born and 162 preterm infants), and between 1970 and 2002, the third ZLS cohort included 327 children of participants of the first ZLS cohort. Since 2019, the participants of the three ZLS cohorts have been traced and invited to participate in a first wave of assessments in adulthood to investigate their current health and development. This article describes the ZLS study protocol and discusses opportunities, methodological and conceptual challenges, and limitations arising from a long-term longitudinal cohort recruited from a study about development in early life. In the future, the ZLS will provide data to investigate childhood antecedents of adult health outcomes and, ultimately, will help respond to the frequent call of scientists to shift the focus of aging research into the first decades of life and, thus, to take a lifespan perspective on aging.

## Introduction

The Zurich Longitudinal Studies (ZLS) are a set of comprehensive studies on child and adolescent growth, health, and development that are currently expanding into adulthood. This article describes the ZLS study protocol and discusses opportunities, methodological and conceptual challenges, and limitations arising from these cohorts; the participants were recruited for this long-term longitudinal study from a previous study about development in early life. In the future, the ZLS will provide data to investigate childhood antecedents of adult health outcomes and, ultimately, will help respond to the frequent call of scientists to shift the focus of aging research into the first decades of life and, thus, to take a lifespan perspective on aging (Sanders, 2016; Moffitt et al., 2017; Williamson and Leroi, 2019).

### Childhood and Adolescence Are Important for Adult Health and Aging-Related Processes

Evidence is accumulating that early life requires consideration when investigating adult health and aging-related processes. In fact, even prenatal life has been shown to be relevant in this regard: for example, adults who were exposed to *in utero* malnutrition during the Dutch famine in 1944/1945 were reported to suffer from higher rates of coronary heart disease, higher levels of stress reactivity, and poorer mental health and premature brain aging (Roseboom et al., 2006; Franke et al., 2018; Van den Broek and Fleischmann, 2019). Moreover, adult outcome is known to be shaped by the socioeconomic and psychosocial environment of childhood. Individuals who grew up in a suboptimal environment have been found to experience poorer health as adults, independent of their current living conditions; this includes an increased risk of various chronic conditions and poorer mental well-being in mid-adulthood (McCrory et al., 2015; Stafford et al., 2015), lower cognitive abilities (Cermakova et al., 2018; Aartsen et al., 2019), more subjective memory complaints (Nishizawa et al., 2019), an increased risk of frailty (Gale et al., 2016), and generally less successful aging (Brandt et al., 2012) in late adulthood. Other studies have focused on how characteristics of the child, most prominently cognitive abilities and personality traits, are related to adult health outcome and aging. Interestingly, lower childhood and adolescent intelligence has been found to predict more advanced biological age in mid- and late adulthood (Schaefer et al., 2015; Stevenson et al., 2019). Potentially, this explains the increased rates of later-life morbidity and mortality in individuals with lower IQ earlier in life (Batty et al., 2007; Schaefer et al., 2015). Furthermore, complex links have been described between personality traits in childhood and adolescence and adult outcome: For example, childhood sociability was found to be related to better subjective well-being and family relationships in mid-adulthood, and better relationships in mid-adulthood and childhood conscientiousness were predictive of a longer life (Kern et al., 2014).

These exemplary findings demonstrate how early life may shape adult health and development and, ultimately, aging-related processes. Appropriate long-term longitudinal studies are required to advance this understanding.

### Examples of Long-Term Longitudinal Studies

Although a comprehensive overview is beyond the scope of this article, this section describes some well-known longitudinal studies, all of which have provided insights into how early life impacts adult health, development, and aging-related processes.

A number of birth cohort studies have been ongoing for decades and accordingly have tracked their participants from early age into adulthood. For example, the MRC National Survey of Health and Development (NSHD), the oldest of several British birth cohort studies, was initiated in 1946 and included more than 5,000 infants. The focus of the study has shifted from an initial interest in the costs associated with pregnancy and birth to how family and environmental factors impact child growth, educational attainment, and cognitive development. As participants have aged, pathways to physical and cognitive aging have become of increasing interest (for a comprehensive cohort profile, see Wadsworth et al., 2006). Currently, the NSHD cohort members are in their eighth decade of life and are still being followed (Kuh, 2016; Kuh et al., 2016). In the early 1970s, the Dunedin Multidisciplinary Health and Development Study enrolled more than 1,000 infants born in the city of Dunedin, New Zealand. They have been tested repeatedly across childhood, adolescence, and adulthood with comprehensive assessments of their health and developmental status at each time-point. Most recently, the 45-year follow-up was completed (Poulton et al., 2015). Although participants have not yet reached old age, the data of the Dunedin study has already contributed to longitudinal aging research by quantifying the pace of aging in mid-adulthood from parameters of biological age (Belsky et al., 2015).

The Fels Longitudinal Study was initiated in Ohio, USA, in 1929 and aimed to describe normal child growth and development. It enrolled 15 to 20 individuals annually over the course of 70 years, either prenatally or at birth, and followed them across life (Roche, 1992). The data continues to provide insights into early-life antecedents of chronic diseases in adulthood (e.g., Sun et al., 2008; Sabo et al., 2017). The Intergenerational Studies (IGS) comprise datasets of three studies (the Berkeley Growth Study, the Berkeley Guidance Study, and the Oakland Growth Study) on typical patterns of child and adolescent development. They were initiated in the late 1920s and early 1930s in California, USA. The studies used different protocols to follow their participants into young adulthood. All assessed comprehensive data on physical, cognitive, and social development. To increase statistical power, the three studies were then merged, and collaborative follow-up assessments of the 500 individuals were added to the dataset in mid- and late adulthood (see e.g., Grimm et al., 2011 for details on the study protocols). The Lothian Birth Cohorts of 1921 and 1936 include individuals who had participated in the Mental Surveys of 1932 and 1947, two Scottish surveys that during the months of June 1932 and 1947 tested the intellectual abilities of all children born in 1921 and 1936, respectively. More than 1,500 individuals residing in Edinburgh and the surrounding area were traced seven decades later and have been assessed repeatedly since to study differences in and causes of cognitive aging (Deary et al., 2012). The Terman Study of the Gifted, another study set in California, included more than 1,000 school-aged children born in the 1910s with an IQ above 135 and assessed their health and development across childhood, adolescence, and adulthood (Frey, 2018). A new team of researchers then linked the archived datasets with publicly available death records of these individuals and, thus, established a lifespan dataset (Friedman and Martin, 2011).

Reviving old studies has remained popular to date: Currently, several research groups are working on identifying former participants of childhood studies and following them up in adulthood. For example, the Louisville Twin Study, a study on child development related to multiple birth status, is currently piloting the assessment of their former members, who are now middle-aged (Davis et al., 2019). Moreover, a cohort of individuals who were placed into infant care institutions in the 1950s in Switzerland are currently being tracked and invited to participate in a study to describe their life stories (Lannen et al., 2021).

The ZLS joins these long-term longitudinal studies in the quest of understanding the importance of early life for later health and development: The ZLS—initiated in the mid-1950s—are a unique set of three cohort studies born two decades apart that assess the development across a range of domains in more than 1,000 individuals. They span the time between birth and the transition to old age and include typically developing individuals and individuals at risk for neurodevelopmental impairments (i.e., born preterm) and dyads of parents and their children. This makes the ZLS highly valuable for assessing early-life antecedents of adult health and aging in different generations.

### The Zurich Longitudinal Studies – a Brief History

#### The International Children's Center (ICC) Coordinated Longitudinal Studies

In the first half of the 20th century, the majority of studies on child health and development were initiated in North America and were largely shaped by the US child welfare movement at the time (Tanner, 1981, 1998). In Europe, the first cohort studies were initiated only after WWII. For instance, in 1951, the Institute of Child Health at the University of London started a multi-disciplinary study on growth and development from birth to maturity under the guidance of the pediatrician Alan Moncrieff (1901–1971). About 2 years later, the Center Internationale de L'Enfance (International Children's Center, ICC), an institution founded in 1950 by the French government and UNICEF, began a similar endeavor in Paris under the direction of Robert Debré (1883–1978). In 1954, these two eminent child health experts joined with the American pediatrician Frank Falkner (1918–2003) to initiate a set of harmonized European longitudinal studies on child health and development: the ICC Coordinated Longitudinal Studies. In the following years, more cohorts were added from Zurich, Brussels, Stockholm and two centers in Africa (Dakar, Senegal and Kampala, Uganda). Falkner contributed broad expertise in longitudinal research as coordinator of the Fels study and became the central figure of the ICC Studies. By 1957, Falkner had moved from the Fels Research Institute in Yellow Springs, Ohio to Louisville, Kentucky. Louisville was the home of the Louisville Twin Study, still regarded as the largest and most comprehensive twin study worldwide (Davis et al., 2019). Subsequently, the Louisville cohort joined the ICC cohorts.

The ICC studies aimed to assess child health and development in various cultures and included various disciplines such as pediatrics, psychology, education, and social sciences. The ICC studies may be considered the first multidisciplinary longitudinal cohort studies on child health and development to include several nations across Europe and Africa. The ICC studies were concluded when the participants reached young adulthood in the mid-1970s; however, some of the involved centers had ceased data acquisition before then.

#### The ZLS as Part of the ICC Coordinated Studies and Beyond

The first principal investigator of the Zurich branch of the ICC studies and the first ZLS cohort (ZLS-1) was the renowned Swiss pediatrician Guido Fanconi (1892–1979), medical director of the University Children's Hospital Zurich, Switzerland. Besides collaborating with the investigator of the other branches of the ICC studies, he was also in close contact with Dr. Marie Meierhofer, the city physician of Zurich, who initiated a study on the health and development of individuals placed in infant care institutions at that time (see Lannen et al., 2021 for details of that study). Fanconi was succeeded in 1962 by pediatric endocrinologist Andrea Prader (1919–2001), who was keenly interested in understanding the growth mechanisms of children and adolescents. Ultimately, Prader was able to turn ZLS-1 into the most successful of all the ICC studies – as the ZLS-1 became the largest cohort to have been followed up from birth to maturity (Tanner, 1981, 1998). In 1974, the developmental pediatrician Remo H. Largo (1943–2020) continued the ZLS and aimed to expand the scope of the studies beyond the understanding of growth mechanisms. Thus, he added two additional cohorts, largely following the initial protocol of ZLS-1: ZLS-2 enrolled children born preterm and at term, and ZLS-3, the Generation Study, enrolled children of the participants of ZLS-1. Although the vast majority of the ZLS-2 and ZLS-3 participants reached young adulthood in the 1990s and early 2000s, the assessments were only completed in 2020, when the youngest ZLS-3 participant turned 18 years old. The data assessed within the scope of these three studies will together be termed ZLS-Childhood. Since 2005, the last author of this article (OGJ) has been the principal investigator of the ZLS.

#### Aims and Selected Findings of ZLS-Childhood

The aims of ZLS-Childhood were (1) to describe developmental trajectories of individual children in the physical, motor, cognitive, and social domains from birth into young adulthood in three independent cohorts born approximately two decades apart; (2) to understand the mechanisms of growth and development through longitudinal analysis; (3) to compare patterns of typical and atypical development (e.g., in preterm children); and (4) to investigate generational effects between parents and their children. Selected findings that have emerged from the ZLS-Childhood datasets to date are presented in the following section.

**Figure 1:**
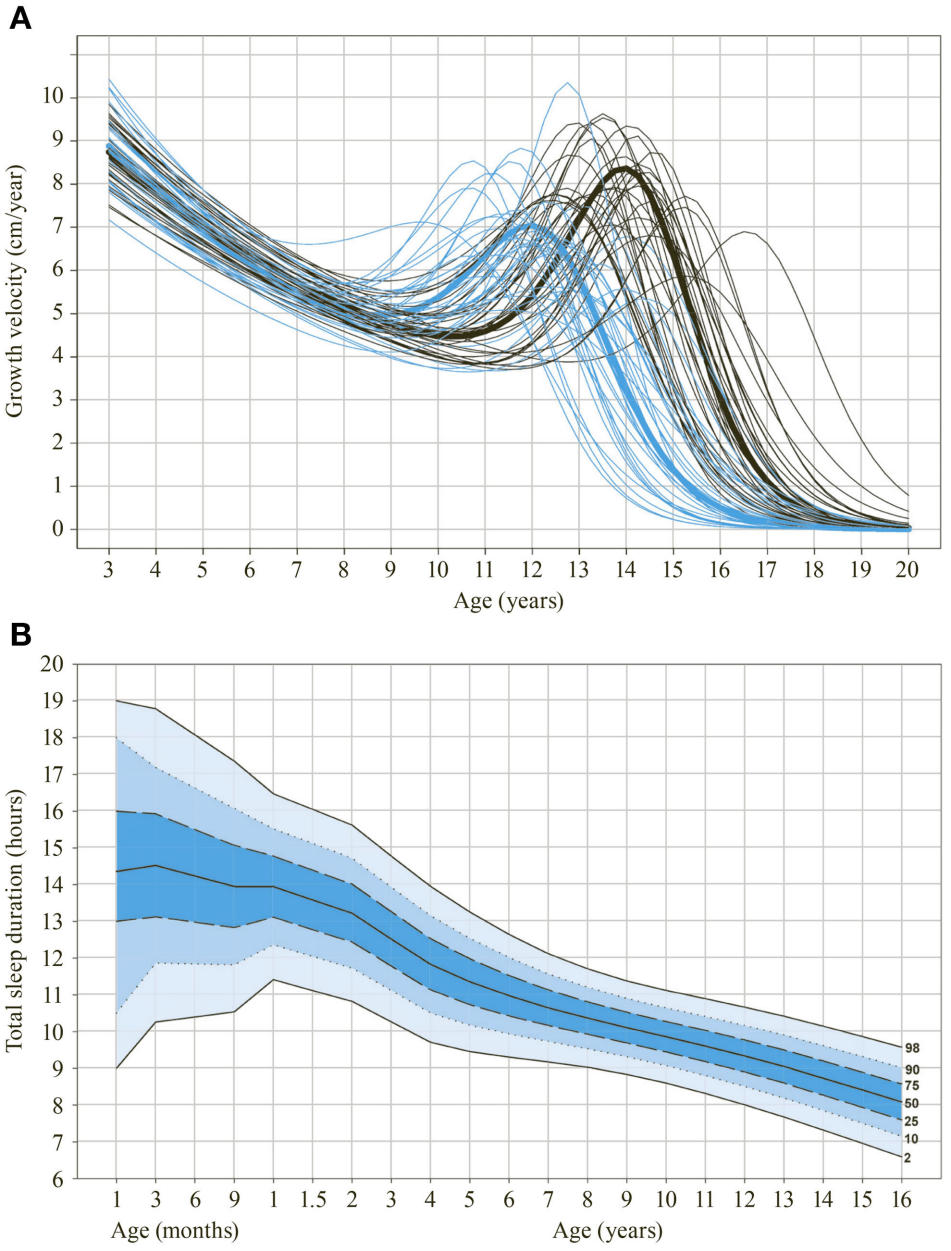
Selected findings derived from ZLS-Childhood data. **(A)** Growth velocity (in cm/year) of individual participants of the first ZLS cohort between 3 and 18 years of age using spline functions and kernel estimation. Blue lines indicate girls, black lines indicate boys. Thick lines indicate group means. **(B)** Reference chart on sleep duration between 3 months and 16 years of age. Data is derived from the second and third ZLS-cohorts (see also Iglowstein et al., 2003).

Intraindividual trajectories of child and adolescent developmental were a key interest of many analyses. For example, the most innovative statistical techniques at that time were applied to understand growth patterns and mechanisms across development; Figure 1A illustrates growth velocity and height acceleration in individual participants of the ZLS-1 using spline functions and kernel estimation (Largo et al., 1978; Gasser et al., 1984). Further, infant motor milestones were found to be poor predictors of neuromotor and intellectual functions at school age and beyond (Jenni et al., 2013), and neuromotor development between early school age and young adulthood was shown to exhibit only moderate stability whereas intellectual abilities and growth variables were more stable (Jenni et al., 2011). Intraindividual developmental trajectories were also described for behavioral variables [e.g., sleep behavior (Jenni et al., 2007)].

Moreover, the development of term- and preterm-born children was compared in detail and, for instance, differences in language development in the first 5 years were described (Largo et al., 1986). Further, comparing the different ZLS cohorts provided insights into the effect of parenting on development: For instance, the timing and intensity of toilet-training changed markedly between the cohorts of ZLS-1 (early and intensive training) and ZLS-2 (later and less intensive training) whereas the development of becoming dry and clean did not differ between the two generations. The conclusion drawn was that the development of bowel and bladder control is a maturational process that cannot be accelerated by early onset and high intensity toilet-training (Largo et al., 1996).

#### Clinical Applications and Further Studies From ZLS-Childhood

Over the years, a number of clinical applications have been developed with ZLS-Childhood data. For example, the personal research interest of the principal investigator Remo H. Largo led to the development of a test battery to assess neuromotor function in clinical practice: the Zurich Neuromotor Assessment (ZNA; Largo et al., 2001a,b). The test battery was recently updated and is applied in the current assessment wave in adulthood (ZNA-2; Kakebeeke et al., 2018). BoneXpert^©^, another frequently used clinical tool, was developed by Danish researchers from the hand X-rays of ZLS-1 participants. This enabled the automatic determination of bone age (Thodberg et al., 2009). Further, reference charts have been published for various developmental domains and continue to inform health care professionals and parents to date [e.g., on growth (Prader et al., 1989), sleep duration—see also Figure 1B (Iglowstein et al., 2003), the Draw-a-Person Test (Jenni, 2013) and play behavior (Bonhoeffer and Jenni, 2018)]. In fact, Zurich Play Behavior is an informal test battery which is used for developmental testing in clinical practice (Bonhoeffer and Jenni, 2018).

In tradition of the ZLS, a number of other comprehensive studies on child development were initiated at the University Children's Hospital Zurich over the past decades. These include the Research and Child Health Outcome (ReachOut) study, a prospective cohort study on children with congenital heart disease who underwent cardiopulmonary bypass surgery (Naef et al., 2019), and a prospective cohort study on children with congenital hypothyroidism (Dimitropoulos et al., 2009).

#### The Future of the ZLS: ZLS-Adulthood and ZLS-Lifespan

The dataset of ZLS-Childhood is very rich but to date, only a fraction of the available data has been analyzed and published. Although analyzing the archived ZLS-Childhood data will provide further insights into child and adolescent development, the main goal of the next years is to expand the studies into older ages and examine how factors in early life contribute to aging-related processes. Thus, the ZLS-Childhood data is currently complemented with data on health and development assessed in adulthood, namely in mid-adulthood and at the transition to old age. These ZLS-Adulthood assessments, eventually, set the basis to initiate a lifespan study, ZLS-Lifespan.

## Methods and Analysis

**Figure 2:**
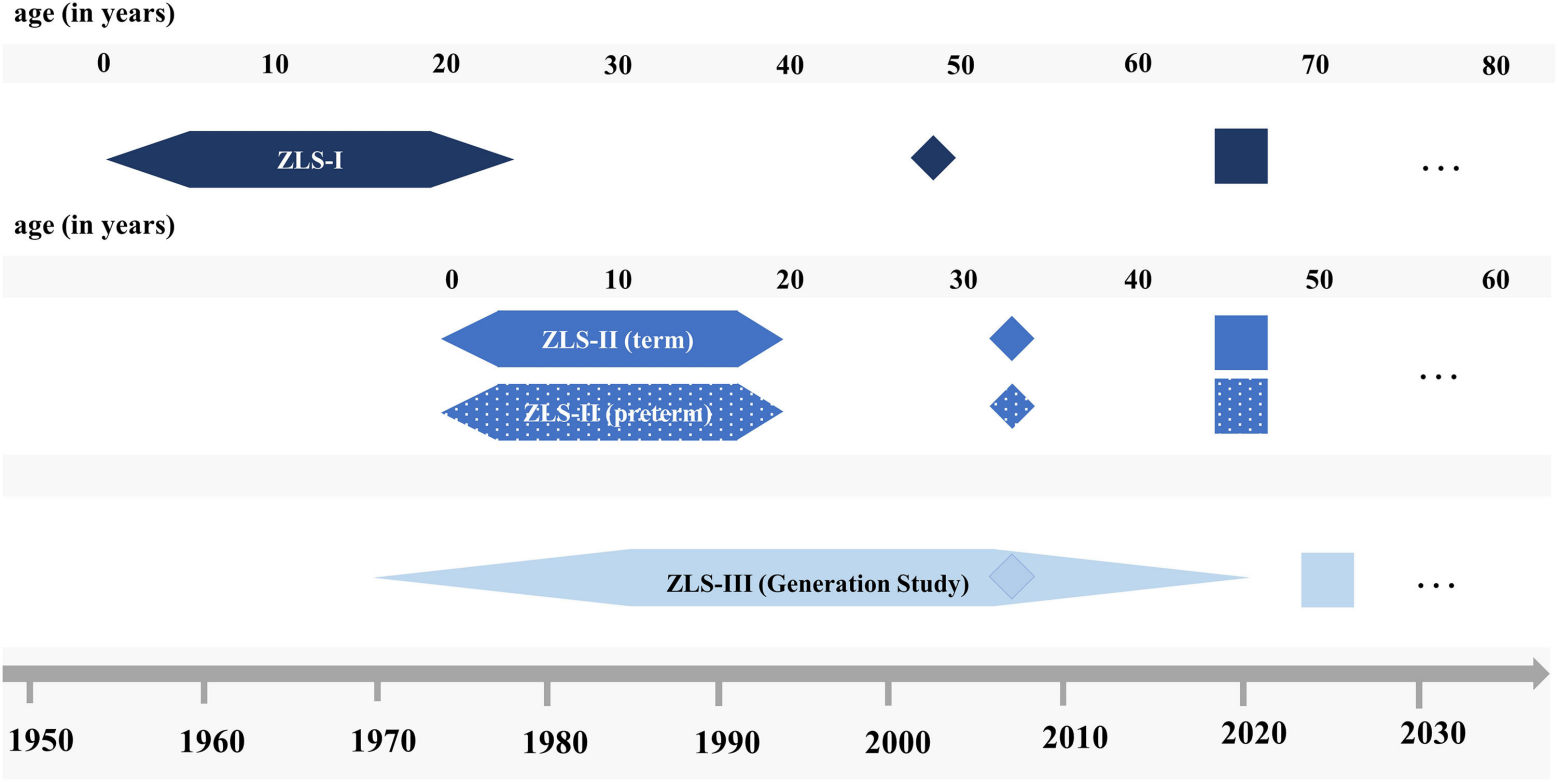
Schematic illustration of the set-up of the three ZLS cohorts. Hexagons indicate ZLS-Childhood assessments, diamonds indicate sub-studies on motor abilities and rectangles indicate the first assessment wave of ZLS-Adulthood. Dots refer to potential future assessments (please refer to text for details).

In the following sections, the recruitment process, study procedure, and assessment protocols are detailed separately for assessments between birth and young adulthood (i.e., ZLS-Childhood) and the first assessment wave in mid- and later adulthood (i.e., ZLS-Adulthood). A final paragraph illustrates how the data will be linked and expanded to form a lifespan data set: ZLS-Lifespan. Figure 2 illustrates the set-up of the three ZLS cohorts.

### Recruitment and Study Procedure

#### ZLS-Childhood

Between 1954 and 1961, a total of 445 infants were enrolled in ZLS-1. They were eligible for the study if they were born into a Swiss family living in the area of Zurich, Switzerland, and were born at the former Women's Clinic of the Cantonal Hospital of Zurich (today the University Hospital Zurich). Eligible parents were approached at random immediately after the birth of their child and informed about the study (Falkner, 1960). If they agreed to participate, they were enrolled, and demographic information was recorded. Because some parents inquired about the enrollment of later-born children into the study, a number of younger siblings were also enrolled at birth.

Between 1974 and 1979, participants for the ZLS-2 were again recruited from the Women's Clinic of the Cantonal Hospital of Zurich and other women's clinics in the Zurich area. A total of 265 infants, 162 born preterm (i.e., before 37 weeks of gestational age) and 103 born at term, were enrolled immediately after birth. Families were eligible if the parents were fluent in German. Parents were approached at random. If parents agreed to participate, the infants were enrolled, and data was collected at the time of birth from preterm and term-born infants and again at term-equivalent age from most preterm infants. Similarly to ZLS-1, some younger siblings were also enrolled at birth.

ZLS-3 enrolled the children of the ZLS-1 participants. After the ZLS-1 participants had reached young adulthood and, thus, had completed their own study participation, they were sent yearly letters inquiring whether they had become parents. If they indicated that they had, they were asked to enroll them in ZLS-3. This resulted in a total of 327 infants of 174 ZLS-1 participants enrolled in ZLS-3 between 1970 and 2002.

In all three cohorts, regular assessments were then scheduled at 1, 3, 6, 9, 12, 18, and 24 months of age and annually thereafter until the age of 9 years. For the remaining duration of the studies, the timing of the visits differed between the cohorts: For ZLS-1, the children were assessed every 6 months (girls from the age of 9.5 years, boys from the age of 10.5 years) until the annual increment in height was <0.5 cm per year. Thereafter, the children were seen annually and finally discharged when the increment in height had become <0.5 cm in 2 years (Prader et al., 1989). For ZLS-2, children were assessed at 10, 14, and 18 years of age onsite and were sent a number of questionnaires at the ages of 12 and 16 years. For ZLS-3, assessments continued annually (except at age 17 years) until children turned 18 years old. For all three cohorts, assessments were scheduled at intervals of ±2 days (1 month assessment), ±1 week (3–18 months assessments), and ±2 weeks (for all remaining assessments) from the birthday of the child (Falkner, 1960). For preterm infants, the assessments were planned at the child's age corrected for prematurity across the entire study period.

**Figure 3:**
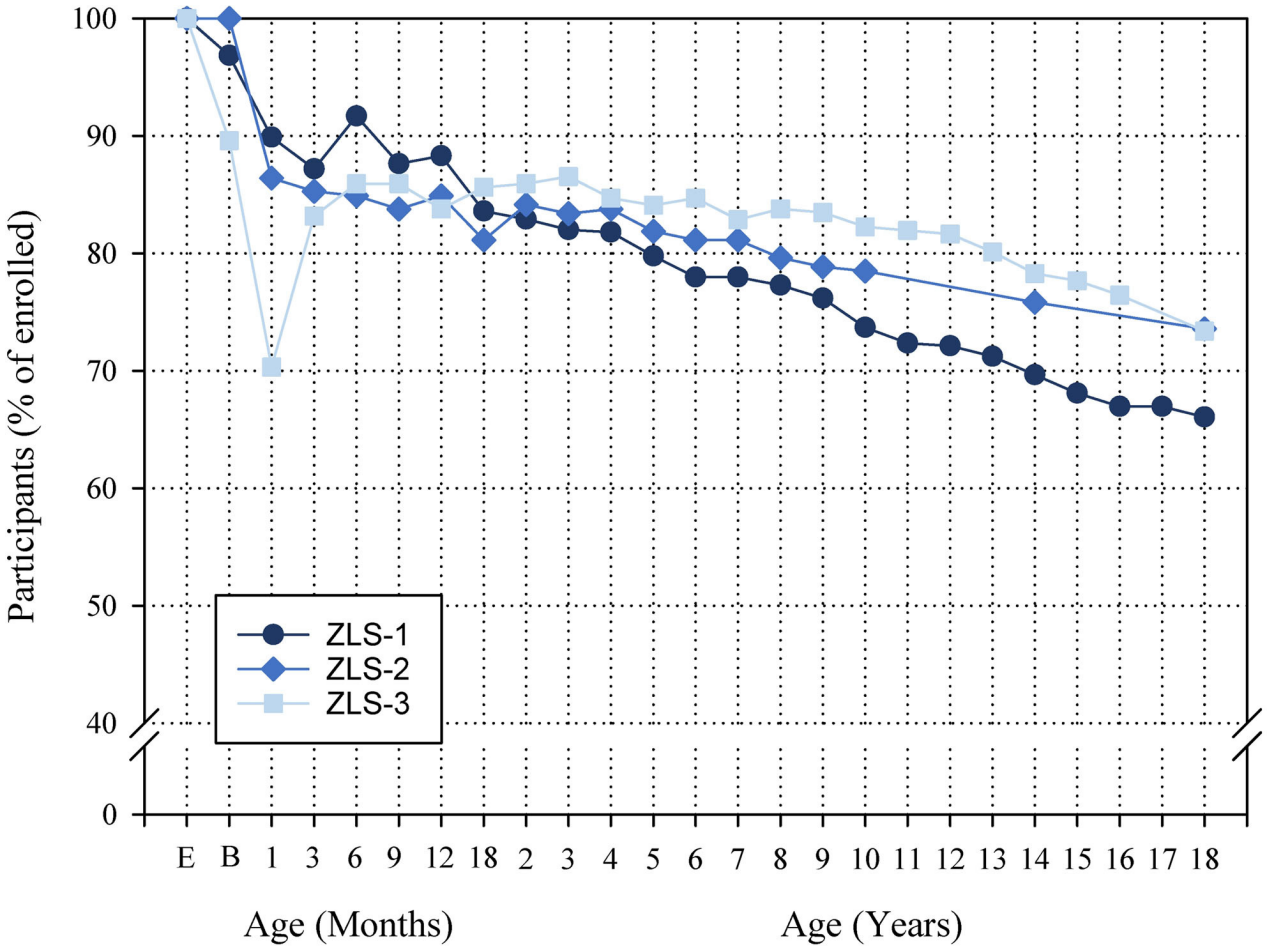
Overview of the number of individuals enrolled in the three ZLS cohorts and the participation rate over the course of the studies. Semi-annual assessments and assessments after 18 years of age in the ZLS-1 cohort are not shown. In the process of digitizing the ZLS-Childhood archives, minor adaptations of these numbers may be necessary if further information on study participants becomes available. E, Enrollment; B, Birth.

Figure 3 provides an overview of the number of individuals who were enrolled in the three ZLS cohorts and how the participation rate developed across childhood and adolescence (note: in the processes of digitizing the ZLS-Childhood archives, minor adaptations of these numbers may be necessary if further information on study participants becomes available). After the initial enrollment, the participation rate dropped markedly because some families failed to participate in any of the assessments (for these infants, only information of enrollment is available; ZLS-1: *n* = 13; ZLS-2: *n* = 0; ZLS-3: *n* =31). Additionally, in the ZLS-2 cohort, the initial drop is partly explained by the fact that 30 preterm and 1 term infants died in the neonatal period. In the ZLS-3 cohort, a number of children were only enrolled at 3 months of age with data on birth being assessed retrospectively. In the ZLS-1 cohort, 2 participants and in the ZLS-3 cohort, 1 participant died during childhood. At the 18-year final assessment common to all three cohorts, of the initially enrolled participants, 66.1% participants of ZLS-1 (*n* = 294), 73.6% participants of ZLS-2 *(n* = 195), and 73.4% participants of ZLS-3 (*n* = 240) attended.

**Figure 4:**
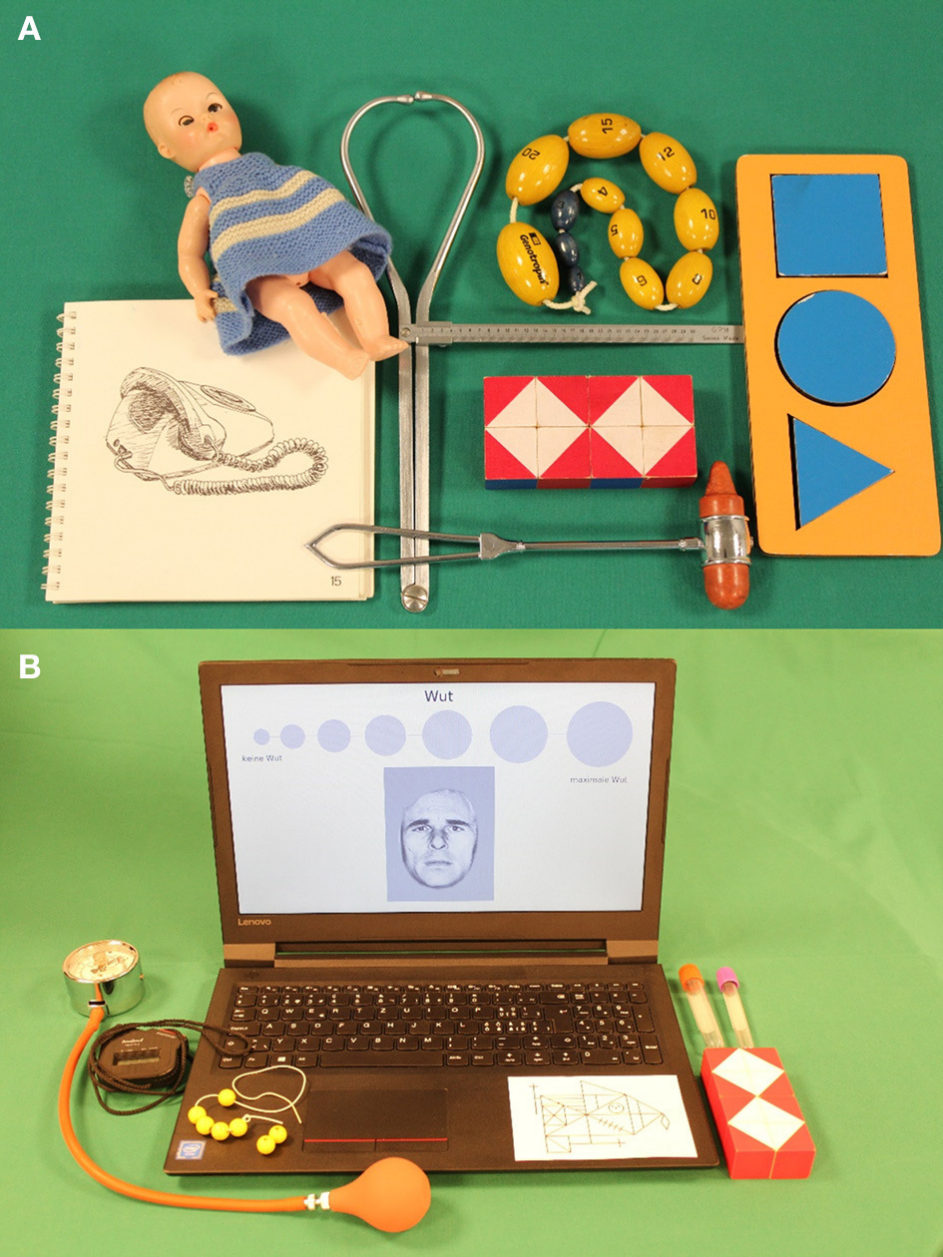
Illustration of example assessment tools used for the ZLS assessments. **(A)** ZLS-Childhood. **(B)** ZLS-Adulthood (first assessment wave).

Study participants were assessed at the former Growth and Development Center (today the Child Development Center) at the University Children's Hospital Zurich. Visits lasted approximately half a day. The children's physical, motor, cognitive, and social health and development was assessed by a pediatrician. Figure 4A illustrates some of the tools that were used. The accompanying parent, mostly the mother, was interviewed by a study coordinator and reported on a variety of aspects of the child and the family (see “ZLS-Childhood” for details of the assessment protocol). Parents were not compensated monetarily, but children received a small gift or a small amount of money when they were older, and travel expenses were reimbursed. Feedback on the developmental and health status of the child was provided to the parents, and in most cases the family physician or pediatrician received a short report.

**Figure 5:**
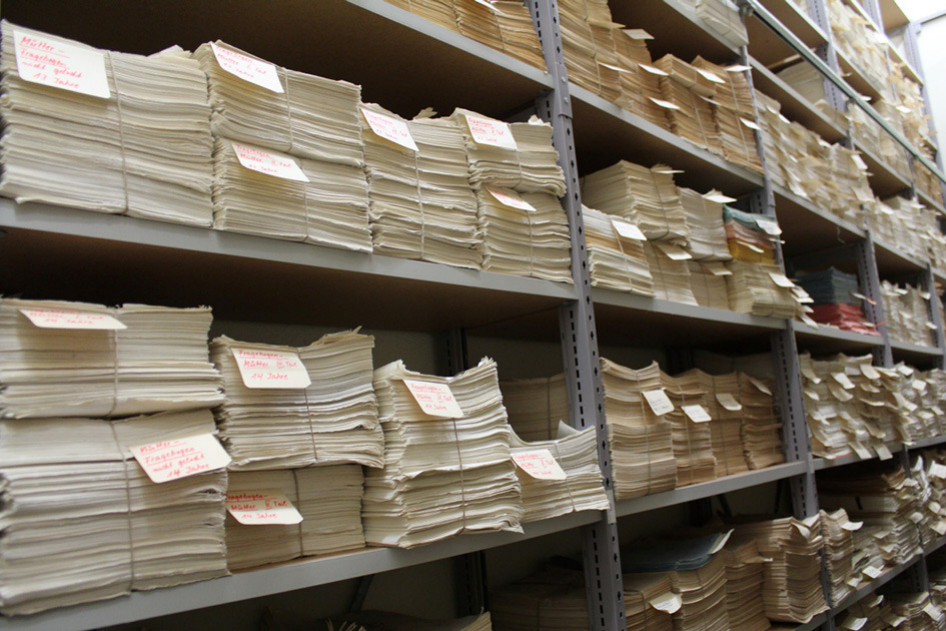
One of the archives hosting the raw data of ZLS-Childhood.

Data was collected on standardized paper record forms and as drawings, analog hand X-rays, and photographic negatives. A large amount of data was subsequently saved as punched card data or on magnetic bands and in some cases, manually typed into Excel files over time. To date, no overarching digital study database has been established. Hard copies of the data and documentation of the study procedure are stored at the University Children's Hospital Zurich, Switzerland. Figure 5 illustrates one of the archives hosting the raw data. Since August 2020, the study data of all ZLS-Childhood waves is being scanned to make digital copies available for data extraction and subsequent inclusion into a study database. Digitization of the ZLS-Childhood data, likely, continues for several years to come. An inventory of the study documentation material is underway.

#### The ZLS Over the Years

After the completion of assessments for ZLS-Childhood in young adulthood, contact with former participants was maintained for some years. Initially, yearly letters were sent to inquire about current contact details, and the study-specific address database was updated accordingly. Participants of the ZLS-1 were further asked whether they had become parents during the past year, and if so, whether they were willing to enroll their children in ZLS-3. Those who did remained closely engaged with the ZLS as their children grew up and were assessed as study participants. At selected time-points, for some of them, information on their own health was assessed as part of the assessment of their children (e.g., IQ assessment of one parent when the participating child was 14 years old). Over the years, a number of events were organized to inform former participants about the study results. Between 2002 and 2004, 98 ZLS-1 participants took part in a substudy investigating the motor abilities of parents of ZLS-3 participants, and in 2007/2008, a subset of ZLS-2 (*n* = 77) and ZLS-3 participants (*n* = 50) took part in an independent study on motor abilities (both unpublished).

The data acquired in ZLS-Childhood was continued to be analyzed, with the main focus shifting from physical development to the development of cognitive and motor abilities (see “Aims and Selected Findings of ZLS-Childhood” for example findings).

In 2014, substantial changes in the legal regulation of research projects with humans came into effect in Switzerland (see “Ethics” for details). As a consequence, all former ZLS participants were contacted by letter and informed about their options to consent to or decline the continued use of their childhood data for future research projects. To achieve this, the study-specific database containing the last-known contact details required comprehensive updating. This was done in two ways. In Switzerland, individuals are registered with their municipality of residence and are required to inform the respective residents' registration office of their new address if they move to a different town. This information is accessible to public institutions, including universities (for details on the legal aspects concerning this search strategy, see Lannen et al., 2021). The current contact details of the majority of individuals were identified in this way, through a number of iterations in case of multiple relocations. A second source of information was other ZLS participants: Alongside the letter with information on the legal changes, participants were sent a return form to indicate whether they knew any other ZLS participants and asked to provide their current contact details. This approach proved fruitful because the cohorts include many pairs of parents and their children (ZLS-1 – ZLS-3) or siblings, particularly within ZLS-3. Information letters were sent to all participants whose current contact details were retrieved (refer to “Ethics” for details on the response options for the continued use of data for future research projects).

The tracing of former study participants is still ongoing, and in the future, the contact details of further individuals are likely to be retrieved (e.g., disclosed by other ZLS participants or through contact with the Swiss Federal Department of Foreign Affairs for participants who emigrated). To date, of the 445 participants enrolled in ZLS-1, 399 participants have been located. Of these, 213 consented and 18 declined consent to the continued use of their data. Of the 265 participants enrolled in ZLS-2, 255 have been located. Of these, 125 consented, and three declined consent. Finally, of the 327 participants enrolled in ZLS-3, 299 have been located. Of these, 173 have consented and one has declined consent (note: individuals who have died either during ZLS-Childhood or since are considered in the number of located individuals but not in the number of individuals who provided or declined consent). For participants who do not reply to the information letter within 2 months, whose current contact details cannot be retrieved despite all efforts, or who have died, the data is retained in the dataset for further analyses (see “Ethics” for details).

Consequently, childhood data of 427 ZLS-1, 262 ZLS-2, and 326 ZLS-3 participants is available for further analyses. Placing the ZLS datasets on a sound legal basis is of utmost importance for the continued use of the data and the recruitment of participants into ZLS-Adulthood, as described below.

#### ZLS-Adulthood

The information on health and development assessed between birth and young adulthood (i.e., ZLS-Childhood) is currently being complemented with additional information in adulthood: At the time of the first assessment wave as described in this article, ZLS-1 participants are approximately between 60 and 65 years old, and ZLS-2 participants are approximately between 40 and 45 years old. The first assessment wave for ZLS-3 has not yet been scheduled.

Individuals who fulfill the following inclusion criteria are eligible for assessments in adulthood: They have participated at least once in a ZLS-Childhood assessment (i.e., those who were only enrolled but never participated are not eligible), their current contact details can be identified, they have not declined continued use of their childhood data for research, and they are able to provide written informed consent for study participation.

Eligible prospective participants are contacted through a letter and invited to participate in the assessment of ZLS-Adulthood. Detailed information is provided about the aim of the study and the study procedure. Participants are asked to contact the study team to schedule an assessment if they agree to participate. If they do not reply, eligible participants are contacted by phone or email a few weeks after the information letter has been sent. Those who cannot be reached after several weeks or for whom no phone number or email is available are sent a brief final letter that highlights the importance of their participation in the study. They are asked to complete and return the enclosed short-form study questionnaire with the signed consent form.

If eligible individuals agree to participate, an assessment is scheduled. Similarly to the assessments during childhood, the onsite assessment of physical, cognitive, motor, and social health parameters lasts about half a day. Figure 4B illustrates some of the tools used for the onsite testing. Assessments are scheduled either in the morning or in the afternoon, whichever is most convenient for the participants. Prior to the onsite assessment, participants complete a questionnaire on their health and well-being, their personality, and their current living situation. They also keep a sleep–activity diary for seven consecutive days prior to the onsite assessment. Participants are asked to participate in specific parts only if they do not want to complete the full assessment protocol (e.g., only the short-form questionnaire).

Onsite testing is conducted in a quiet room specifically designed and equipped for this study in a venue provided by a private foundation (see “Acknowledgments”) by research staff extensively trained in neuropsychological and motor testing. This provides optimal conditions for standardized assessments. Participation is compensated with CHF 125.- for onsite testing and questionnaire and CHF 25.- for questionnaire only, and travel expenses are reimbursed. Participants receive a summarized written feedback on their assessment if they wish. The recruitment of the ZLS-1 and ZLS-2 cohorts was initiated in April 2019, and assessments are planned to be completed within 2 years. However, assessments were suspended for 3 months due to restriction measures issued by the Swiss authorities to halt the spread of the Coronavirus disease 2019 (COVID-19) pandemic. The assessment of the ZLS-3 cohort is planned for after the completion of ZLS-1 and ZLS-2 assessments.

The data is entered and stored in a study-specific electronic case report form implemented in Redcap (Harris et al., 2009, 2019). Video recordings and photographs are saved on a server at the University Children's Hospital Zurich. Hand X-rays are taken at a private health clinic close to the onsite testing venue and stored on a server at the University Children's Hospital Zurich. Blood samples are drawn at the onsite testing venue and analyzed and stored at the University Children's Hospital Zurich. Due to logistic reasons, these measures are not always taken at the same time-point during the assessment. The exact time of the assessment is recorded to take potential effects of timing into consideration for further analyses as appropriate. Both measures are voluntary subparts of the assessment protocol. For individuals who have deceased, the time and nature of death is recorded.

All participants are being informed about and consent to the consolidation of the databases of ZLS-Childhood and ZLS-Adulthood into one joint database: ZLS-Lifespan.

### Assessment Battery

#### ZLS-Childhood

The cataloging and preprocessing of the archived ZLS-Childhood datasets is an ongoing process, and thus, no final overview of the data is presented here. Instead, the structure of the assessment batteries applied in the three cohorts is described, and examples of the instruments used are provided. Details on the changes and adaptations of the assessment protocols within the individual cohorts over the course of the studies and the overlap and differences between the three cohorts are detailed.

The general architecture of the study was the same for all three ZLS-Childhood cohorts: The participants were tracked regularly and at frequent intervals from birth to young adulthood to capture the rapid developmental changes across the first two decades of life. Further, all three studies assessed detailed aspects of the child's proximal and distal psychosocial environment. Table 1 illustrates the domains assessed. The study team gladly provides details on specific parameters that were traced across childhood and adolescence as they become available in the course of the ongoing cataloging, preprocessing and digitization of the ZLS-Childhood data; readers are encouraged to contact the authors. Notably, the developmental domains and environmental factors that were assessed in ZLS-Childhood largely overlap with those suggested more recently as requiring consideration when establishing longitudinal birth cohort studies (see Golding, 2009a for details). This highlights the foresight of the ZLS researchers at the time. They understood the importance of considering the central drivers of long-term development, which remain important to the present.

**Table 1:** Overview of assessed domains of health and development during ZLS-childhood.

**Physical health and development**
Pregnancy and birth
Neonatal development
Illnesses and accidents
General physical health status
Anthropometry
Neurology
Hearing and vision
Pubertal development
Bone age
Dental status
Orthopedic information and podogram
Bowel and bladder control
Feeding and nutrition
Sleep
**Motor development**
Motor milestones
Fine and gross motor abilities
**Cognitive development**
General intellectual abilities^a^
Visuo-motor abilities
Language development
Academic abilities
**Social development**
Attachment, parent-child interaction
Emotional development
Psychosocial development and integration
**Environment**
Socio-economic status
Living situation
Parenting behavior
Critical life-events

*The study team gladly provides details on specific parameters that were traced across childhood and adolescence as they become available in the course of the ongoing cataloging, preprocessing and digitization of the ZLS-Childhood data; readers are encouraged to contact the authors*.

a *See Figure 8 for detailed information on how intellectual abilities were assessed across childhood and adolescence in the three ZLS-cohorts*.

**Figure 6:**
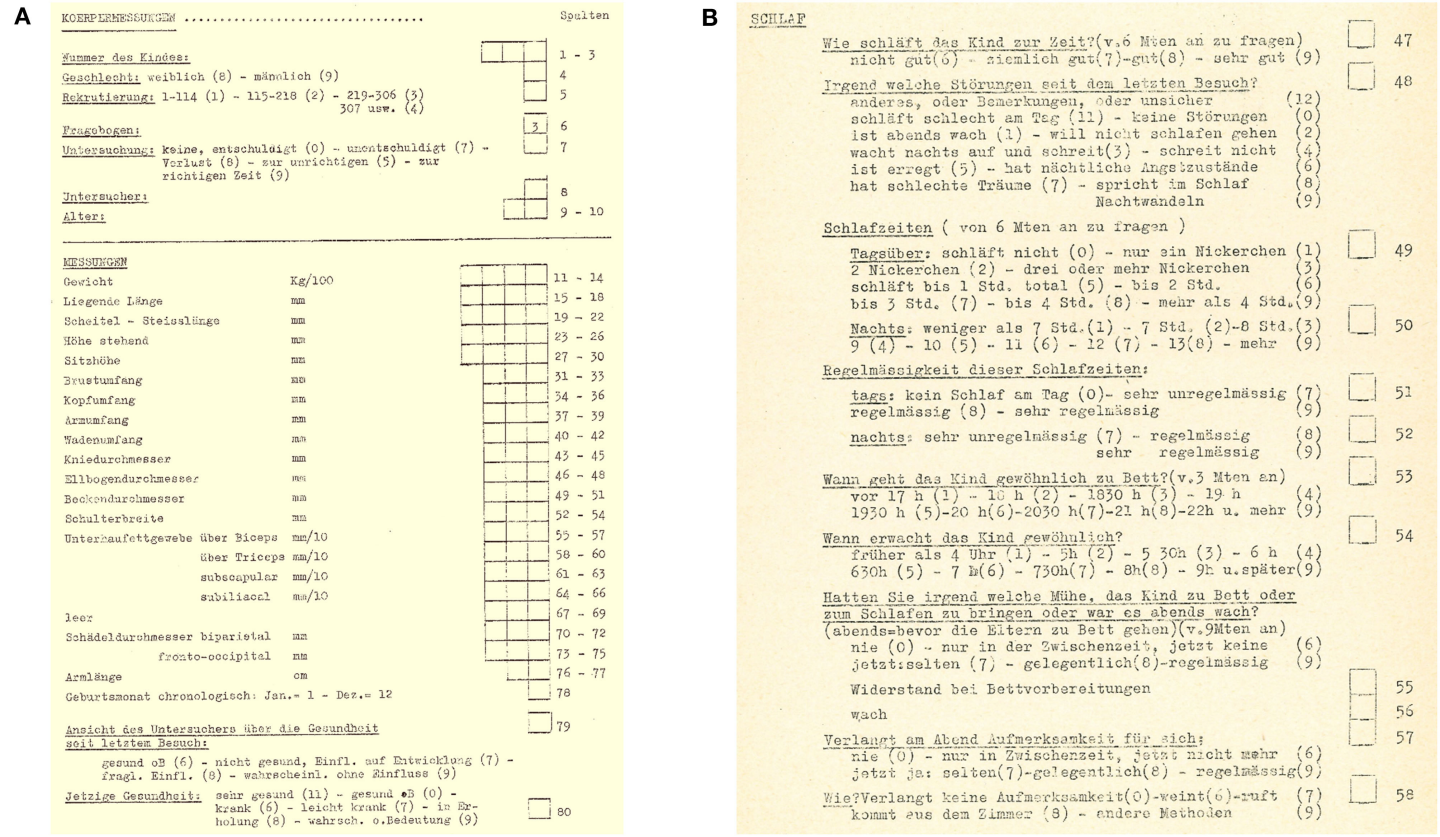
Illustration of standardized record forms used for all three cohorts during ZLS-Childhood. **(A)** Assessment of anthropometric measures. **(B)** Assessment of sleep quantity and quality (see Supplementary Material 1 for English translation).

**Figure 7:**
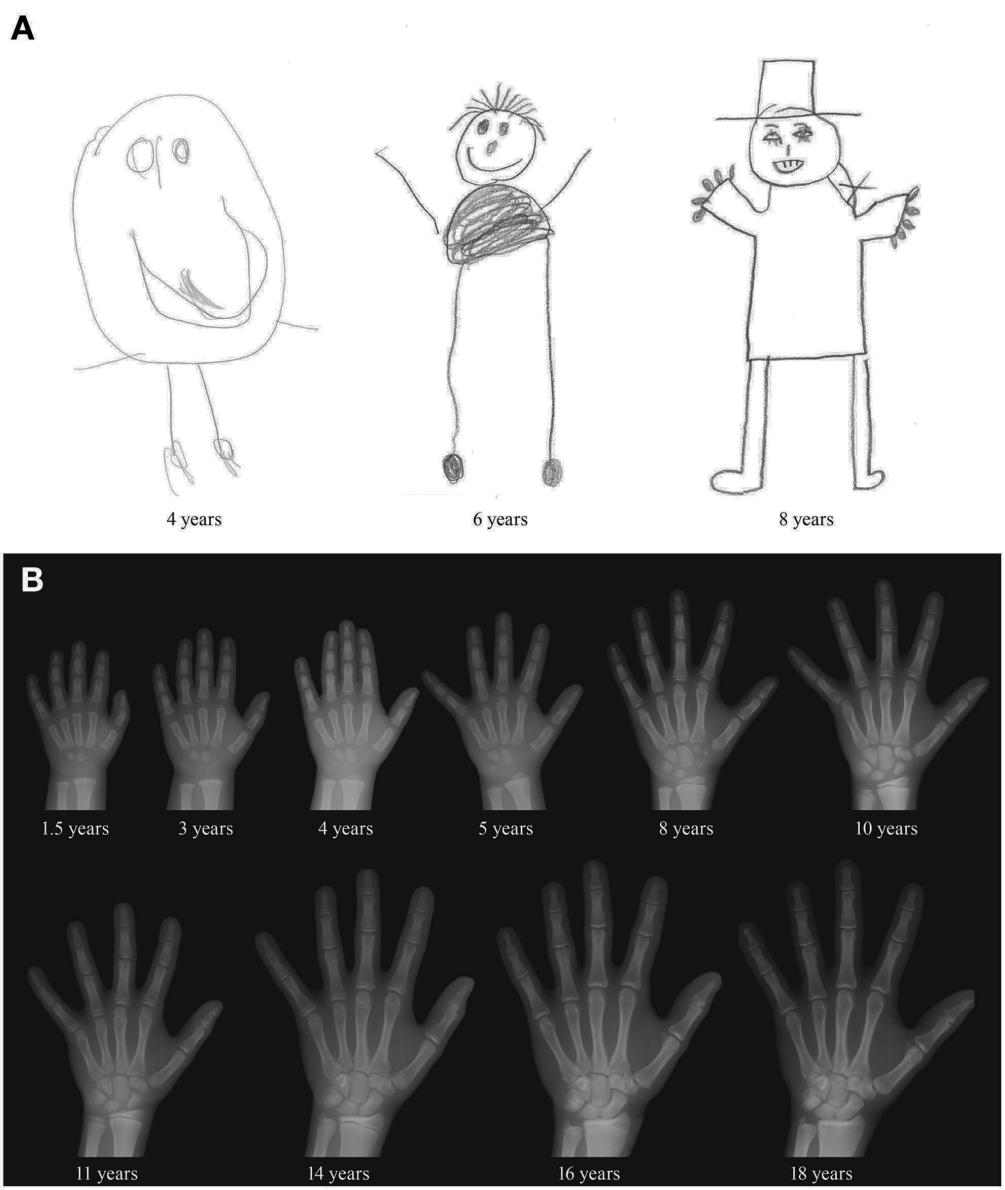
Illustration of drawings and hand X-rays. **(A)** Draw-a-Person-Test of one male participant at 4, 6, and 8 years of age. **(B)** Serial hand X-rays of one male participant.

The study design and assessment instruments were initially decided upon within the context of the ICC Coordinated Longitudinal Studies (see “The Zurich Longitudinal Studies – A Brief History” for details on the ICC studies), of which the first ZLS cohort was part. Subsequently, they were largely retained for ZLS-2 and ZLS-3. The instruments were primarily quantitative in nature and included standardized physical, motor, and cognitive examinations by pediatricians and structured interviews with the parents. Examinations were documented on standardized record forms (see example for anthropometric measures in Figure 6A; Supplementary Material 1 for English translation). Parents reported on child health (e.g., illnesses and accidents that had occurred since the previous visit), child development (e.g., sleep and eating behavior) and environmental factors (e.g., sociodemographic variables) by answering questions with specified response options. Figure 6B illustrates this standardized format for the assessment of sleep behavior (Supplementary Material 1 for English translation). At specific time-points, teachers reported on child personality, behavior, and development through questionnaires sent by mail. Projective tests [e.g., the Rosenzweig Picture Frustration Study (P-F Test) (Rosenzweig et al., 1948), the Draw-a-Person Test (Goodenough, 1926; Harris, 1963)], open-ended questions to the parents and teachers (e.g., “What are the child's strengths?,” “What are the child's difficulties?”) and unstructured notes by the examiners and study coordinators complemented the quantitative assessments. Children prepared drawings at various ages (Figure 7A), and hand X-rays to assess bone age (Figure 7B) and full-body photographs to document the development of the physical appearance were taken at various time-points. Although the potential importance and value of collecting biological material in longitudinal cohort studies has been noted recently (Jones, 2009), this was not done with ZLS-Childhood participants except for the unsystematic collection of teeth from a subset of ZLS-2 and ZLS-3 participants.

**Figure 8:**
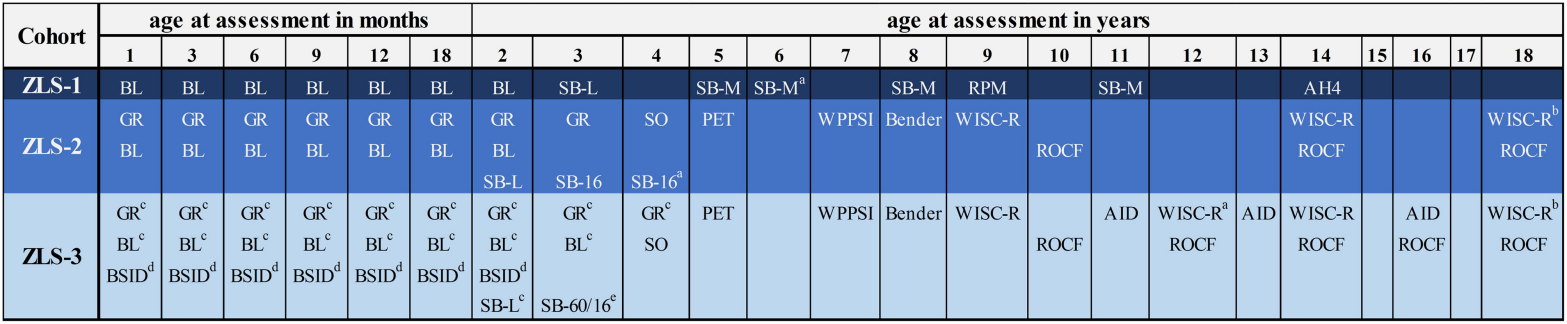
Instruments to assess cognitive abilities in ZLS-Childhood. AH4: AH4 Group Test of General Intelligence (Heim, 1970). AID: Adaptives Intelligenz-Diagnostikum (“Adaptive Intelligence Diagnostics”) (Kubinger and Wurst, 1985). Bender: Bender's Visual Motor Gestalt Test (Clawson, 1962). BL, Brunet-Lézine Test (Brunet and Lézine, 1951); BSID, Bayles Scales of Infant Development (Bayley, 1969); GR, Griffiths Test (Griffiths, 1954); PET, Psycholinguistischer Entwicklungstest (German adaptation of the “Illinois Test of Psycholinguistic Abilities”) (Angermaier, 1974); RPM, Raven's Progressive Matrices (Raven, 1947, 1956); ROCF, Rey-Osterrieth Complex Figure (Rey, 1941); SB-L/SB-M, Stanford-Binet Test of Intelligence L-Form/M-Form (Terman and Merrill, 1937; Lückert, 1957); SB-16, Stanford-Binet Test of Intelligence 1916-version (Terman, 1916); SB-60, Stanford-Binet Test of Intelligence 1960-version (Terman and Merrill, 1960; Terman et al., 1965); SO, Snijders-Oomen Non-Verbal Intelligence Tests (Snijders-Oomen, 1977); WISC-R, Wechsler-Intelligence Scale for Children-Revised (German version; Tewes and Titze, 1983; Willich and Friese, 1994). ^a^Applied only in a small subgroup of study participants. ^b^Selected subtests only (Similarities, Vocabulary, Digit Span, Picture Arrangement, Block Design, Object Assembly). ^c^Tests were omitted from the assessment protocol over the course of the study (i.e., approx. after the 140th participants). ^d^Task was only introduced in the course of the study (i.e., approx. after the 120th participant). ^e^Until 1983: 1916-version was applied, after 1984: 1960-version was applied.

Within the individual studies, assessment instruments were retained from birth into young adulthood if possible and reasonable. For example, the questions in the parental interviews on the current health status of the child remained the same over time, the assessment of anthropometric measures was repeated at every visit in the same way, and sociodemographic data was assessed repeatedly with an equivalent form. Questions and tests were added, adapted, or removed as developmentally appropriate. For example, questions related to breast-feeding were complemented and finally replaced by questions on general nutritional habits (e.g., intake of vegetables) as the child got older. Similarly, standardized tests were chosen to accurately estimate cognitive and motor abilities at specific ages (see Figure 8 for an illustration of the tests applied to assess cognitive abilities at different ages).

The assessment instruments overlapped considerably between the three cohorts. In particular, the structured interviews with parents to assess the current health status, various aspects of child development, and factors of the psychosocial environment remained the same across all three studies. In addition, the longitudinal assessment of physical development across childhood, which was a primary interest of the researchers who initiated the ICC studies, remained an integral part of the assessment protocol in ZLS-2 and ZLS-3. Accordingly, parameters of physical development were documented in the same way across the three cohorts (e.g., anthropometric measures, see Figure 6A). Retaining the same instruments across the three cohorts ensured comparability of data and allows the investigation of developmental patterns between generations (see “Aims and Selected Findings of ZLS-Childhood” for an example of the comparison of parenting practices over time).

Despite this general overlap in the assessment protocols between the three cohorts, a number of adaptations were implemented over time to integrate advances in research and technology or for other reasons.

The frequency of assessments was reduced in the second and third cohorts compared to the first: In ZLS-1, more than 30 assessments were conducted in most children. Visits were scheduled at half-year intervals during puberty to track growth patterns. Importantly, this allowed the growth spurt in adolescence to be described (Largo et al., 1978; Gasser et al., 1984). It was considered that such an intense assessment protocol was no longer essential with the ZLS-2 and ZLS-3 cohorts to answer questions about physical development. Consequently, the semi-annual assessments, and in the ZLS-2 cohort also a number of yearly assessments, were omitted from the assessment protocol.

Some of the assessments of physical development were adjusted or omitted from the study protocols of the ZLS-2 and ZLS-3 cohorts. To describe pubertal development, children's physical appearance in ZLS-1 was documented by taking naked photographs from different angles. From this data, the Tanner stages were derived and pubertal development quantified (Tanner, 1962). Ethical considerations related to children's privacy led to the suspension of taking naked photographs in ZLS-2 and ZLS-3. Instead, children were photographed in underwear and later, full-body photographs were omitted altogether.

Cognitive development was assessed with standardized tests in all three cohorts, but the specific tests at particular ages differed between the cohorts and, in some cases, even between participants of one cohort. Reasons for the replacement of specific tests included the availability of updated or more appropriate tests or the personal preferences of the principal investigator at the time. For example, in the ZLS-1 and ZLS-2 cohorts, in accordance with the protocol of the ICC studies, the Brunet-Lézine test of infant development (Brunet and Lézine, 1951) was applied in all participants for the assessments between 1 and 24 months of age. This test provides information on four domains—posture, oculo-manual coordination, language, and socialization—and it summarizes general infant development as a developmental quotient. In the ZLS-3 cohorts, the Brunet-Lézine test was used initially but was later replaced with the Bayley Scales of Infant Development (BSID; Bayley, 1969). The BSID provides separate estimates for mental and psychomotor development.

New assessment instruments were also introduced with advancements in technology. For example, a computerized tapping task to assess motor abilities was applied with the ZLS-2 and ZLS-3 cohorts.

In summary, the data that is available for the three cohorts incorporated in ZLS-Childhood provide a detailed and comprehensive description of child and adolescent development. The study protocols have been designed to allow the investigation of a multitude of research questions, and the data was carefully recorded and archived. Nonetheless, their detailed understanding and processing requires time, money, and creativity. It has been noted that the time spent to acquire fresh data in a primary data collection study may be equivalent to the time spent understanding old, archived data (Jones, 2010). Future publications using the ZLS datasets to study child and adolescent development or to investigate how factors at the beginning of life impact adult outcome will have to detail how the childhood data was processed and made useable for such analyses.

#### ZLS-Adulthood

The assessment protocol of the first wave of ZLS-Adulthood is equivalent for participants of the ZLS-1 and the ZLS-2 cohorts. Potential adaptations for the assessment of ZLS-3 participants will be kept minimal to ensure comparability between the three cohorts. The assessment battery for ZLS-Adulthood was designed to include several practical and theoretical considerations. In childhood, development was assessed across a number of domains, including physical, mental, motor, and social domains. Following this precedents, the instruments for ZLS-Adulthood sought to capture indicators of health and development across a range of domains. Further, the assessment battery was intended to incorporate the understanding of health “as a state of physical, mental and social well-being and not merely the absence of disease or infirmity” as defined by the World Health Organization (WHO; World Health Organization, 1948). Thus, alongside instruments for assessing physical and mental symptoms, instruments were included to gauge physical, mental, and social well-being and quality of life. In the future, the datasets of the ZLS seek to provide information about how developmental factors contribute to aging-related processes later in life. To establish a basis for this, the WHO's definition of “healthy aging” was considered: Healthy aging describes the process of developing and maintaining functional abilities, that is, intrinsic capacities such as physical and mental abilities and characteristics of the proximal and distal living environment of individuals (World Health Organization, 2016). Consequently, a number of physical and mental abilities such as executive functions were assessed and detailed information on the environment was sought (e.g., social relations). Further definitions of “healthy aging” (see e.g., Aronson, 2020 for a review), that include aspects such as satisfaction with life and well-being were also considered. Lastly, in recent years, reports have provided evidence that aging-related processes may be more closely related to biological age than to chronological age (e.g., Belsky et al., 2015; Horvath and Raj, 2018). Aligning with this research, biological markers that allow the quantification of state-of-the-art aging parameters were added to the assessment protocol.

Tables 2 and 3 provide an overview of the assessment instruments employed in ZLS-Adulthood. They were selected because (i) they had been applied as part of previous assessments in childhood and thus allowed intraindividual longitudinal comparison of performance [e.g., Rey-Osterrieth Complex Figure, (Rey, 1941)]; (ii) they are established clinical tools, enabling the comparison of results with clinical populations [e.g., S-words (Aschenbrenner et al., 2000)]; (iii) they have previously been applied in the Swiss Household Panel (Voorpostel et al., 2016) or the Swiss Health Survey (Bundesamt für Statistik, 2014), two large, representative studies in Switzerland, and thus, their results may be compared with the general Swiss population [e.g., socio-demographic questions, Satisfaction with Life Scale (Glaesmer et al., 2011)]; (iv) they have previously been described in the literature as sensitive to aging-related changes [e.g., dual task walking (Beurskens and Bock, 2012); grip strength (Cooper et al., 2011)]; or (v) they are being used in other long-term longitudinal cohort studies on health and development [e.g., in the Dunedin study, (Poulton et al., 2015)].

**Table 2:** Test battery of onsite assessment for the first wave of ZLS-Adulthood (grouped by domain).

**Domain**	**Assessment instrument**	**Order of assessment**^**a**^
**Physical health**		
Audiometry	Interacoustics audiometer (AD629)	
Blood pressure and heart rate	Boso-Carat professional	
Anthropometric measures	Portable stadiometer (Seca 213), scale (Seca)	16
Visual acuity	Snellen Eye Test, Lang-Stereo Test	
Lung function	CareFusion MicroLab spirometer	
Grip strength	Martin Vigorimeter	
Blood sample	–	na^b^
Hand X-ray	Standardized X-ray of left hand	na^b^
**Motor abilities**		
Fine motor abilities	Zurich Neuromotor Assessment – 2 (Kakebeeke et al., 2018)	3
Pure motor abilities	Zurich Neuromotor Assessment – 2 (Kakebeeke et al., 2018)	11
Balance	Zurich Neuromotor Assessment – 2 (Kakebeeke et al., 2018)	18
**Cognitive abilities**		
Verbal comprehension	Similarities (Petermann and Petermann, 2012)	7
Perceptual organization	Block Design (Petermann and Petermann, 2012)	13
	Matrices (Petermann and Petermann, 2012)	14
Alertness	Tests of Attentional Performance (TAP), (Zimmermann and Fimm, 2009)	1
Processing speed	Coding (Petermann and Petermann, 2012)	25
	Symbol Search (Petermann and Petermann, 2012)	24
Working memory	Digit Span (Petermann and Petermann, 2012)	5, 22^c^
Fluency	Phonetic (S-Words) (Aschenbrenner et al., 2000)	21
	Semantic (Animals, Fruits) (Aschenbrenner et al., 2000)	4
	Design (Regard et al., 1982)	8
Cognitive flexibility	Trail Making Test (Strauss et al., 2006)	9
Inhibition/Interference	Stroop (Strauss et al., 2006)	23
	TAP Go/No-Go (Zimmermann and Fimm, 2009)	2
Planning	Tower Task (Unterrainer et al., 2019)	26
Verbal memory	Hopkins Verbal Learning Test (Brandt and Benedict, 2001)	12, 15^d^
Visuo-spatial memory	Rey-Osterrieth Complex Figure (Rey, 1941)	6, 10^e^
**Further assessments**		
Dementia screening	Montreal Cognitive Assessment (Nasreddine et al., 2005)	20
Dual-Task Walking	Subtracting 7 from 250 while walking (Abernethy, 1988); automatic assessment of gait parameters with Optogait (Lienhard et al., 2013)	19
Emotion perception	Computerized emotional rating task (Chiu et al., 2018)	27
Photograph	Nonstandardized and standardized facial photograph and full-body photograph	17
Current mood	Multidimensional Mood Questionnaire (Steyer et al., 1997)	28

*The assessment protocol is equivalent for participants of the ZLS-1 and the ZLS-2 cohorts. Potential adaptations for the assessment of ZLS-3 participants (initiated after recruitment of ZLS-1 and ZLS-2 participants has been completed) will be kept minimal to ensure comparability between the three cohorts*.

a *Tasks are applied in fixed order (1 = assessed first). Tasks 1 through 18 (and 28) are part of the short-form assessment protocol*.

b *Due to logistic reasons, these measures were not always taken at the same time-point during the assessment. The exact time of the assessment is recorded to take potential effects of timing into consideration for further analyses as appropriate; both measures are voluntary subparts of the assessment protocol*.

c *12: Digit Span forward/backward, 22: Digit Span sequential*.

d *Learning and recall/recognition condition*.

e *Copy and delayed recall condition*.

**Table 3:** List of questionnaires completed by participants during the first wave of ZLS-Adulthood assessments.

**Topic or construct**	**Questionnaire**	**Abbrev**.	**Included in short form**
**General information about yourself**			
Socio-demographic information	Questions based on the SHP and SHS or study specific: Education, current working and living situation, income, civil status, children	–	yes (less detailed)
General information about personal life	Questions based on the SHP and SHS or study specific: Leisure activities, political and religious engagement	–	yes
**Your health**			
General perception of own health	Question of the SHS	–	yes
Information on current health	Questions based on the SHP and SHS or study specific: Drinking, smoking, drug consumption, medication intake, current illnesses, support measures (e.g., hearing aid)	–	yes
Information on previous illnesses and accidents	Based on the questionnaires applied as part of ZLS-Childhood	–	yes
Health-related quality of life	Short Form Health Survey (Morfeld et al., 2011; Wirtz et al., 2018)	SF-12	yes
Handedness	Edinburgh Handedness Inventory – Short Form (Veale, 2014)	EHI	no
Physical activity	Based on the questionnaires applied as part of ZLS-Childhood and on the SHP and SHS	–	yes
Diet	Based on the questionnaires applied as part of ZLS-Childhood and a questionnaire of the “Swiss Society for Nutrition”	–	no
Sleep quantity and quality	Pittsburgh Sleep Quality Index (Hinz et al., 2017)	PSQI	yes
Subjective memory complaints	Memory Complaint Questionnaire (Crook et al., 1992)	MAC-Q	no
**Your life**			
Biographical transitions	Study specific questions: Age at and appraisal of different biographical transitions and critical life events (e.g., puberty, transition to parenthood, serious illness, unemployment,…)	–	yes
Sense of Coherence	Sense of Coherence L9 (Schuhmacher et al., 2000)	SOC-L9	yes
**Your well-being**			
Life satisfaction	Satisfaction with Life Scale (Glaesmer et al., 2011)	SWLS	yes
Psychological Wellbeing	Ryff Inventory (Ryff and Keyes, 1995; Staudinger et al., 1999)	–	no
Domain-specific satisfaction	Study specific questions, partly based on the SHP and the PFB-K: Evaluation of satisfaction with various life domains (e.g., health, work, personal relationships…)	–	yes
Beliefs about Aging	Essentialist Beliefs About Aging Scale (Weiss et al., 2016)	–	no
Mental health	Mini-Symptom-Checklist (Franke, 2017)	Mini-SCL	no
**Describing your personality**			
Personality traits	NEO-Five-Factor Inventory – 30-item short form (Körner et al., 2008)	Neo-FFI-30	yes
Empathy	Saarbrücker Persönlichkeits-Fragebogen (based on the Interpersonal Reactivity Index) (Davis, 1983; Paulus, 2011)	SPF/IRI	no
**Coping with difficult situations**			
Resilience	Resilience Scale – 13-item form (Schuhmacher et al., 2000)	RS-13	yes
Basic psychological needs	Incongruence Questionnaire (Grosse Holtforth and Grawe, 2003)	–	no
Cognitive Emotion Regulation	Cognitive Emotional Regulation Questionnaire (Garnefski et al., 2001; Loch et al., 2011)	CERQ	no
Self-Efficacy	General Self-Efficacy Scale (Beierlein et al., 2012)	–	yes
**Your relationships**			
Attachment behavior	Adult Attachment Scale – Revised (Schmidt et al., 2016)	AAS-R	no
Attachment style	Adapted from Relationship Scales Questionnaire (Bartholomew and Horowitz, 1991)	RS-Q	yes
Attachment to Parents	Retrospective 1-item assessment (Neumann, 2002)	–	yes
Loneliness	6-Item Scale for Overall, Emotional, and Social Loneliness (Gierveld and Tilburg, 2006)	–	no
Social Support	Social Support Questionnaire (Fydrich et al., 2007)	F-SozU	yes
**Additional information**			
Evaluation of study participation	Study specific questions	–	yes
Impact of COVID-19 pandemic^a^	Study specific questions to assess the impact of the Covid-19 pandemic on the lives of study participants (Supplementary Material 2)	–	yes
Sleep–activity diary^b^	Study specific diary on sleep (at night and naps during the day) and activity (e.g., sports, alcohol consumption) to be completed in the mornings and evenings for seven consecutive days.	–	no

*The assessment protocol is equivalent for participants of the ZLS-1 and the ZLS-2 cohorts. Potential adaptations for the assessment of ZLS-3 participants (initiated after recruitment of ZLS-1 and ZLS-2 participants has been completed) will be kept minimal to ensure comparability between the three cohorts. Questionnaires are grouped thematically and presented after the respective sub-headings. PFB-K, Partnerschaftsfragebogen Kurzform (Relationship Questionnaire Short Form) (Kliem et al., 2012); SHP, Swiss Household Panel (Voorpostel et al., 2016); SHS, Swiss Health Survey (Bundesamt für Statistik, 2014)*.

a *These questions were added to the questionnaire in June 2020*.

b *The diary is not part of the questionnaire but completed on paper by all participants*.

The onsite physical examination and neuropsychological and motor tests are applied in fixed order. This allowed the definition of a short protocol by dividing the full protocol into two parts (see Table 2). It also ensures similar cognitive load between the encoding and recall trials of memory tasks across all participants. The examination starts with a task to assess initial alertness [i.e., Tests of Attentional Performance, Alertness subtest; (Zimmermann and Fimm, 2009)]. Breaks are taken at fixed time-points during the assessment and at the request of participants. The assessment may be distributed across two appointments if this is more convenient for the participants. The start and end times of assessments are documented to later investigate potential effects of daytime on performance. The full protocol lasts between 4 and 6 h and the short protocol approximately 2 h, including breaks. During the COVID-19 pandemic, measures have been implemented to ensure a safe environment for participants and staff. This includes the installation of a plexiglass wall to shield participants from staff for all assessments at the table and wearing of face masks for all other tasks if the mandatory distance cannot be kept.

The study questionnaire is subdivided into several thematic parts (see subheadings in Table 3). For the short form, instruments or individual items are omitted from each part, but the general order is retained. Participants receive a link to the online questionnaire implemented in RedCap (Harris et al., 2009, 2019) or are sent a paper version, whichever is most convenient for them. Paper questionnaires are entered manually into the database by the study staff. Completion time is between 90 and 120 min for the full version and approximately 45 min for the short form. In the course of the COVID-19 pandemic, the questionnaire has been expanded by a number of items to inquire about the impact of the pandemic and the measures taken by the authorities to halt its spread on the lives of the study participants (see Supplementary Material 2). This will help to systematically quantify a potential impact on health and well-being over the course of the data collection.

### ZLS-Lifespan

Linking the rich datasets of ZLS-Childhood with the comprehensive data on health and development assessed during the first wave of ZLS-Adulthood allows the investigation of how early life impacts adult outcome. By adding additional waves of ZLS-Adulthood in the future, a ZLS-Lifespan dataset will develop and eventually provide information about health and development across the lifespan. A number of considerations will support this process. Firstly, the ZLS datasets need to be placed on a solid ethical and legal basis, including considerations related to making data available for other researchers [see e.g., the Concordat on Open Research Data (Concordat on Open Research Data, 2016)]. The effort to collect written informed consent from participants for the continued use of their childhood data (see “Ethics” for details), to have them consent to the assessment of further data in adulthood, and to link these datasets is currently ongoing. Secondly, the individual data storage units need to be replaced by one common database that allows childhood and adulthood data to be linked and is readily expandable in the future. The digitization of the ZLS-Childhood data has been initiated in August 2020 by scanning all paper forms. In the course of the next years, this data will be extracted from the digital copies and merged in a common data base with the data assessed as part of the first wave of ZLS-Adulthood (e.g., in RedCap). Efforts are undertaken to ensure consistent terminology and detailed documentation in this process. Thirdly, to assess aging-related processes and lifespan trajectories, further assessment waves need to be added to the dataset. Proper planning is required in this regard, including timely grant acquisition and keeping in touch with eligible study participants between assessment waves. Currently, yearly newsletters are sent to study participants to inform them about the progress and the future plans of the ZLS. Updated study protocols to describe the methods of future assessment waves will be published as appropriate. Similarly, updates have been published previously for long-term longitudinal birth cohort studies such as the NSHD cohort (Kuh et al., 2011) and the Lothian birth cohort study (Taylor et al., 2018).

It has been noted that it requires multiple generations of researchers to establish long-term longitudinal and ultimately lifespan studies (Mroczek et al., 2011). Accordingly, the efforts of the researchers who initiated and nurtured the ZLS over decades to improve the understanding of child health and development will now be continued by others.

### Data Analysis

To embrace the multitude of data that has been assessed over the course of more than half a century will require a number of statistical methods, some of which are proposed here. Regression models will be considered to assess how factors of health and development in the first two decades of life may impact adult health outcomes. To this end, the variables measured across child and adolescent development will be considered potential predictors. Candidate variables will be specified depending on the research question. Analyses will be begun by employing classical regression models (i.e., linear regression for continuous outcomes and logistic regression for binary outcomes). When appropriately combined, the number of potential predictors available through the comprehensive ZLS-Childhood datasets may be reduced to increase their reliability and potentially also their relevance. For example, some indicators measured many times during childhood and adolescence (e.g., estimates of intellectual abilities) could be summarized by individual trajectories, typically via random intercepts and random slopes estimated from generalized linear mixed models [of which growth curve models are a particular case (Verbeke and Molenberghs, 2000)]. With this, autocorrelation can be accommodated to take into account dependencies induced by the repeated measurements in a longitudinal study (Diggle, 2002). Other examples include assessments of multidimensional domains such as motor abilities, which are measured through dozens of variables. This information may be summarized in components using the technique of simple component analysis (e.g., Rousson and Gasser, 2004). The summaries thus obtained, instead of the original variables, may subsequently be used as predictors in the regression models.

Further, neural networks may be used for data analyses because many predictors will be available even after data reduction. Neural networks are flexible models that allow the identification of complicated interactions among various predictors (Cheng and Titterington, 1994; Magoc and Magoc, 2011). Exploring the outputs of such models may provide suggestions for interactions to be included in the classical regression models.

Ultimately, which analyses are most suitable will be decided by the specific research question. Previous similar endeavors to study development from childhood into adulthood may guide these choices (e.g., McArdle et al., 2009; Friedman et al., 2010; Jones and Peskin, 2010). By employing appropriate statistical approaches, the ZLS datasets, provide a rich source of data that may help to answer questions about how health develops across the lifespan.

### Ethics

Over the course of a long-term longitudinal study, ethical considerations and legal regulations are likely to change and thus require continuous monitoring (Birmingham and Doyle, 2009). In fact, the legal basis for the ZLS has changed several times since their initiation. When they started, no approval by a formal ethical committee was required for such studies in Switzerland. Parents were informed about the aims and the procedure of the study, that participation was voluntary, and that they could withdraw at any time. They provided oral consent for participating in the study with their child. At the beginning of every visit, parents were informed about the planned assessments. All ZLS-Childhood assessments were conducted according to this procedure.

In 2008, the Ethical Committee of the University Children's Hospital Zurich classified the ZLS as unproblematic (*Unbedenklichkeitsbescheinigung*; Certificate of Clearance). This allowed the analyses of the data assessed as part of ZLS-Childhood to continue. In 2014, the new Federal Act on Research involving Human Beings, Human Research Act (HRA; The Federal Assembly of the Swiss Confederation, 2014) came in force in Switzerland. This required the ZLS to be evaluated by the Ethical Committee of the Canton of Zurich, Switzerland. The approval for the procedure with the ZLS-Childhood data (detailed below) and the assessment of the first wave of ZLS-Adulthood was granted in 2018 (Basec-Nr. 2018-00686). Amendments for future waves of assessments will be submitted as required.

As a consequence of the new HRA coming into force in 2014, the participants of ZLS-Childhood are contacted by letter and informed about what the legal basis of the ZLS had been when they were initiated and how the new HRA impacts the continued use of the ZLS-Childhood datasets for future research projects. Participants are explained the two types of ZLS-Childhood data: The vast majority of the data is considered “coded,” i.e., the data is linked to the specific person via a code (HRA Art. 3; The Federal Assembly of the Swiss Confederation, 2014). This concerns for example the data on physical growth (see Figure 6A) or on cognitive development. In contrast, some data—most prominently the photographs—contain identifying information and are, thus, considered “uncoded.” Participants are asked to return the signed consent form and indicate whether they provide (1) full consent for the continued use of their data, i.e., the use of coded and uncoded data for projects of the University Children's Hospital Zurich and the sharing of coded data with external institutions (without sharing of the respective code) or (2) partial consent, i.e., limiting the use of the data to either research projects of the University Children's Hospital Zurich (coded and uncoded data) or to external institutions (coded data). Participants are further asked to contact the principal investigator (OGJ) if they decline to consent to the continued use of their data and are sent a confirmation letter if they do. Their data is then removed from the ZLS archives, and they are not contacted any further. Participants are further informed that the Ethical Committee of the Canton of Zurich has issued a consent surrogate in case of no reply within 2 months after participants having received the information letter. This allows the continued use of the data for future research projects. The surrogate consent also covers the data of individuals who have died during the study or since or of those whose current contact details cannot be retrieved despite all efforts (HRA Art 34; The Federal Assembly of the Swiss Confederation, 2014). All future research projects with ZLS-Childhood data require individual ethical approval.

In the course of the process described above, several individuals have declined to provide consent for the continued use of their data (see “The ZLS Over the Years” for the detailed numbers). Although some have done so without further comment, others have voiced serious criticism of the way the ZLS-Childhood data was assessed and, more generally, how children were treated as study participants in the 1950s and 1960s. In particular, the naked photographs and the detailed physical examinations to assess pubertal development were described as traumatic by some individuals. The current study team takes this criticism very seriously: Former study participants are offered a personal dialogue with the principal investigator (OGJ) and their feedback is forwarded to the Ethical Committee of the Canton of Zurich, Switzerland to ensure independent documentation. A detailed discourse on the role of the children in the ZLS is needed to transparently describe the setting in which the ZLS-Childhood data, particularly of the ZLS-1 cohort, was collected and to acknowledge the distress the study has caused some of the participants. Thus, a historical and ethical reappraisal is intended of research and parenting practices and the treatment of and attitude toward children in the 1950s and 1960s.

Individuals who participate in the first wave of ZLS-Adulthood provide written informed consent for participating in the assessment, the continued use of the respective data and the linkage of the ZLS-Adulthood data with ZLS-Childhood data.

## Discussion

It has been argued that “longitudinal data are perhaps the most valuable type of data in adult development and aging research” (Mroczek et al., 2011, p. 128). The current article illustrates how the ZLS, previously described as among the most complete studies on child development worldwide (Tanner, 1998), are currently expanding into adulthood. The resulting long-term longitudinal dataset allows the investigation of how early life impacts adult health outcome and, ultimately, aging-related processes.

Long-term longitudinal studies provide a number of opportunities and challenges, some of which are discussed here in the context of the ZLS. This article will conclude by elaborating on some potential research questions that may be approached within the ZLS dataset.

### Opportunities of Longitudinal Studies to Investigate the Impact of Childhood on Aging

Intraindividual trajectories of health and development over very long periods of time can only be studied with long-term longitudinal studies. Noteworthy examples in this regard are the Terman study, which followed individuals from mid-childhood until death (Friedman and Martin, 2011), and the NSHD, a birth cohort study whose members are currently 70 years old (Kuh et al., 2016). With the first wave of ZLS-Adulthood, comprehensive data will become available on health and development from birth into mid-adulthood (ZLS-2 and ZLS-3 cohorts) and to the transition to old age (ZLS-1 cohort). The timespan assessed will gradually increase as ZLS-participants age and additional waves are added. In addition to spanning several decades, the study design of the ZLS has other unique properties: The three cohorts include individuals born more than two decades apart, individuals with and without risk for neurodevelopmental impairments (i.e., born preterm or at term), and dyads of parents and children. This makes the ZLS highly valuable for assessing early-life antecedents of adult health and aging.

In prospective studies, data is assessed at or close to the events it relates to. For example, in the ZLS, health indicators were assessed at every visit. Parents reported on illnesses and accidents only for the period since the last visit (i.e., mostly for the past year)—a period that may be remembered well. Such prospectively collected information has been suggested to be more accurate than information that is recalled later in life (Golding, 2010). Arguably, this is particularly true when investigating the impact of early life on adult health outcomes: the period between when an event happened (i.e., childhood) and when it is remembered (i.e., old age) may span several decades.

It is important to keep in mind that long-term longitudinal studies are “semiarchival”: Data assessed at earlier time-points becomes archival even if new waves of data are continuously collected (Mroczek et al., 2011). This methodological peculiarity comes with a number of challenges and limitations that are addressed next.

### Challenges and Limitations of *Archived* Part of Longitudinal Datasets

Understanding the design and procedure of a study that was planned and conducted by other researchers and whose data was not originally intended to be used for what it is now is a major challenge, and accordingly, it requires time to immerse oneself into the study's dataset (Jones, 2010; Pienta and Lyle, 2018). Documentation may be spotty (Jones, 2010), and the information needs to be reviewed and inventoried carefully (Pienta and Lyle, 2018). The documentation of the three ZLS cohorts is scattered across scientific publications (e.g., Falkner, 1960), countless notebooks, and hand-written correspondence among members of the study team and external collaborators. Studying these documents has already and will continue to support the process of understanding the design of the ZLS. Furthermore, former members of the ZLS study team have previously consulted or still continue to support the current team [e.g., the former director of the Growth and Development Center of the University Children's Hospital Zurich (RHL), the former study coordinator (EK), and the former study statistician (LM)]. This likely clarifies some of the gaps in the written documentation. Nevertheless, some thoughts and decisions made by the previous study teams may not be reproducible, and the corresponding data needs to be treated accordingly in future analyses: for example, labeled as missing or to be interpreted with care.

When expanding studies on child and adolescent development into adulthood, it is important to keep in mind that the study design and the assessment instruments were decided upon with other research questions in mind than those of interest now. Consequently, information that would be desirable to have may not be available from the dataset. For example, the ZLS participants were enrolled at birth. Consequently, only a limited amount of data is available on the prenatal environment. However, a number of recent studies have reported that prenatal factors may considerably impact health in later life and aging-related processes (e.g., Franke et al., 2018). The detailed information on the medical history of pregnancy provided by the parents and the early life anthropometric variables which are available in the ZLS datasets provide some information on prenatal development. Further, the comparison of preterm and term-born individuals (ZLS-2 cohort) can inform about how the respective environment during the last part of pregnancy impacts later health outcome. However, for a detailed analysis of prenatal factors, other datasets may be better suited and should be used instead. It has been noted that prenatal enrollment in prospective cohort studies is difficult (Golding, 2009b). Thus, studies that have succeeded in doing so (e.g., the Generation R Study; Jaddoe et al., 2006) should be carefully curated, so participants can be followed into adulthood.

Alongside the enrollment criteria, which may not have been ideal to study the impact of early life on later life and aging, the instruments, likely, were not chosen with a long-term longitudinal study in mind either. For example, processing speed has been suggested as an important mediator of changes in cognition throughout the lifespan (e.g., Salthouse, 1996; Nettelbeck and Burns, 2010; Gilsoul et al., 2019). However, the ZLS-Childhood dataset contains very limited assessments of processing speed. Consequently, this cannot be studied with the ZLS datasets.

It is important to recognize and acknowledge the limitations and shortcomings that are inherent in the archived datasets. Making them transparent can help to manage the expectations about what can be investigated (e.g., Wadsworth et al., 2006).

### Challenges and Limitations of *Expanding* Datasets Into Long-Term Longitudinal Data

Researchers who aim to expand a study on child development into a long-term longitudinal and eventually even lifespan study are confronted with both methodological and conceptual challenges and limitations. Methodologically, first and foremost, the study sample is predefined. The number and the type of participants (e.g., representative population sample vs. specific clinical sample) are determined by the originally enrolled sample.

Two strategies have been described when deciding upon the number of participants enrolled in a longitudinal cohort study (Golding, 2010): Including a very large number of individuals but not a large amount of data, the “huge-and-thin” strategy, or including fewer individuals of whom in-depth data is assessed, the “small-and thick” strategy. A good example of the latter is the Dunedin study, an ongoing cohort study that has included approximately 1,000 individuals who are assessed very comprehensively and at frequent intervals (Poulton et al., 2015). Similarly, the ZLS enrolled a few hundred children in each of the three cohorts, jointly accounting for approximately 1,000 study participants. Although a high follow-up rate is always of importance in long-term longitudinal studies, this is particularly true for relatively small studies such as the ZLS to ensure a valid sample size. The first wave of ZLS-Adulthood assessments began with an event to which former participants were invited. Members of the study team informed the audience about previous results and announced the upcoming assessment wave. This social event resonated well with the study participants, and similar events are planned for the future alongside yearly newsletters. As ZLS-Childhood is expanded by ZLS-Adulthood and, ultimately, will develop into ZLS-Lifespan, it is important to build on previous experiences to maintain participation into older age and ensure optimal follow-up. The NHDS team has taught a number of valuable lessons in this regard (Kuh et al., 2016).

Besides the number of potentially eligible participants, representativeness of the study sample is important for the generalizability the findings. First, the initial sample should be representative of the population that it represents. In the ZLS-1 and ZLS-2 cohort, the research teams recruited children born into Swiss or German speaking families residing in the area of Zurich. It is reported that parents were approached at random (Falkner, 1960). Unpublished descriptive analyses of the ZLS-1 study team suggest that the cohort was representative of the population of Zurich with regard to paternal occupation; however, this remains to be evaluated in more detail. Publicly available census data (Federal Statistical Office, 2017) will be used to clarify representativeness for the ZLS-1 and ZLS-2 cohorts. The ZLS-3 cohort was recruited from the children of the ZLS-1 participants and representativeness needs to be assessed with regard to the respective population of ZLS-1 participants. Another concern in long-term longitudinal studies is whether study participants remain representative of the original sample: Selective study drop-out is a well-known issue (Young et al., 2006; Launes et al., 2014) and was discussed already in the context of the ICC studies in the 1960s (Falkner, 1960). Sociodemographic, health, and other participant characteristics determine who remains in the study over time. In the ZLS, participants to be assessed in adulthood are likely not a representative subset of those individuals enrolled at birth. This may limit generalizability of the results, and potential bias needs to be considered when interpreting future results. The data from assessments prior to drop-out help to quantify a potential shift in representativeness, for instance toward a study sample with higher socioeconomic status. Further, publicly available representative population datasets help in this regard: It has been suggested that data from household panels may serve as useful reference point for the general population (Siedler et al., 2011). Accordingly, the ZLS-Adulthood study questionnaire incorporates a number of questions from the Swiss Household Panel (Voorpostel et al., 2016) and the Swiss Health Survey (Bundesamt für Statistik, 2014), two large datasets assessed in a representative population sample in Switzerland. With these, characteristics of ZLS-Adulthood participants can be compared to characteristics of the general population. This will help to describe the ZLS cohorts in the future.

It has been noted that one of the major challenges in longitudinal studies is to balance continuity of measurements with keeping abreast of latest standards, and researchers are likely to implement what is current in their period (Hofer and Piccinin, 2009). In an attempt at appropriate balance, the study protocol of ZLS-Adulthood was designed to retain the general architecture of ZLS-Childhood: to assess parameters of physical, motor, cognitive, and social health and development and to closely describe the proximal and distal environment of participants. Some of the methods that were employed for ZLS-Childhood assessment were maintained for ZLS-Adulthood [e.g., standardized measures to assess anthropometric information, hand X-rays to quantify bone health, and the Rey-Osterrieth Complex Figure to assess visuospatial constructional abilities and memory (Rey, 1941)]. Some methods applied in ZLS-Childhood have been substituted by more modern ones, but the underlying construct remains identical. For example, duration and quality of sleep was previously assessed with a number of individual items (illustrated in Figure 6B). These were replaced in ZLS-Adulthood by the well-validated and clinically established Pittsburgh Sleep Quality Index (Hinz et al., 2017). Finally, a number of new methods have been added to the study protocol. For example, blood samples were collected for the first time. This allows the investigation of parameters of physical health (e.g., glycated hemoglobin, HbA1c) and, possibly even more interestingly, the quantification of biological age. Recently, a number of different approaches to do so have been published, and biological age is increasingly recognized as a reliable indicator of aging-related processes (Belsky et al., 2015; Horvath and Raj, 2018). This illustrates how advances in research may have an effect on the protocol of a long-term longitudinal study.

Importantly, not only are the ways of assessing particular constructs likely to change over the course of a long-term longitudinal study but also the constructs themselves. In the ZLS, some constructs were only formally introduced into the study protocol recently as part of the assessment of ZLS-Adulthood. Very likely, however, data touching on similar or overlapping constructs has already been assessed across childhood and adolescence without those labels. For example, the concept of executive functions was first defined only in the 1970s, but a “control mechanism” was discussed in the mid-19th century (Goldstein et al., 2014). Accordingly, the interviews with the parents of the first ZLS cohort (initiated in the 1950s) included a number of questions related to self-control and self-regulation, for instance on the frustration tolerance of the child, on strategies the child employs to self-sooth, and on the child's reactions to altered routines. Interestingly, similar questions are currently included in the preschool version of the Behavior Rating Inventory of Executive Functions (BRIEF-P; Daseking and Petermann, 2013). However, unlike in such psychometrically validated rating scales, the relevant questions in the ZLS datasets were neither labeled as relevant to executive functions nor arranged as specific scales; rather, they are scattered across many different records. If they are identified and combined appropriately (see below for examples of how other researchers have previously approached this issue), they may reliably assess the underlying construct and serve as potential predictor of adult health outcome. In fact, child self-control has been shown to predict adult physical health, substance dependence, personal finances, and criminal offending outcomes in the Dunedin study (Moffitt et al., 2011).

Innovative methods are needed to extract psychometrically sound constructs from existing archival data of long-term longitudinal studies. A number of researchers have presented ways of accessing old data with new concepts. For example, Jack Block developed the California Q-Sort (CQS) method to derive a valid measure of personality from the diverse interview and observational data assessed in the IGS (Block, 1961). His comprehensive book provides details of the methods (Block, 2008). To date, his measure is used to study personality development across life (e.g., Jones and Meredith, 1996; Chopik and Grimm, 2019; Chopik et al., 2019) and how personality traits early in life impact health in adulthood (e.g., Peskin and Jones, 2015). Recently, one study has even derived a new construct from the CQS personality measure: a subset of items thought relevant to empathy was selected from the original measure through correlational analyses and based on theoretical considerations. Then, the newly developed measure was validated in a contemporary undergraduate student sample against an established rating scale of empathy, the Interpersonal Reactivity Index (Davis, 1983). With this, the authors were able to investigate longitudinal changes in empathy across the lifespan (Oh et al., 2020). In the comprehensive ZLS datasets, a number of such unlabeled constructs are likely buried, and they need to be identified to become available for future analyses.

Even if the underlying construct is defined, the tools to assess them were unlikely to have been constant over time (see Figure 8 for how cognitive abilities were assessed at different ages in ZLS-Childhood). Researchers have advanced statistical methods to cope adequately with this issue. For example, in the IGS datasets, item response theory and latent curve modeling were combined in a longitudinal growth model to bring different measures of vocabulary and short-term memory into a common scale (McArdle et al., 2009). Thus, these constructs became accessible despite them being assessed with various age-appropriate tools over time. Similar approaches may be employed to investigate developmental trajectories in the ZLS datasets.

In summary, the ZLS will not constitute a final, static dataset for a while. Instead, the datasets will continue to develop as researchers engage in the tasks of working with the archived raw data to develop appropriate measures for constructs of interest and link them to adult outcome. A number of potential research questions that can be answered with the ZLS datasets in the future is outlined below.

### Potential Questions to Be Addressed Within the Scope of the ZLS

Presumably, an infinite number of research questions could be addressed with the ZLS datasets. Some of those can be directly translated into a hypothesis, others may be approached in a data-driven and possibly, even exploratory, manner. The benefits of the complementary use of these strategies has been discussed previously (e.g., Kell and Oliver, 2004; Elliott et al., 2016; Matsumuro and Miwa, 2019).

Potential questions include these: How stable are motor and cognitive abilities between early childhood and late adulthood? Does a constitutional delay or an acceleration of puberty have a long-term impact on health and development in adulthood? Is the association between motor and cognitive abilities in adulthood stronger than their relatively weak association in childhood? Do physical activity and motor abilities in early childhood impact health in later adulthood? What are the childhood factors that contribute to cognitive decline in aging? How do personality traits in childhood impact adult health and development? Are early developmental milestones in social behavior predictive of adult outcome? How are emotional and social development linked to resilience over the lifespan? Are individuals with advanced biological age in childhood also biologically older in adulthood? Do better cognitive abilities in childhood protect against biological aging in adulthood? What are the long-term effects of preterm birth on health, wealth, and well-being? Are preterm-born adults biologically older than term-born adults? Is aging accelerated after preterm birth? Is the decrease in motor skill variability between individuals in childhood followed by an increase in older age? How do different parenting practices in the 1950s and 1970s impact individuals' long-term development? Is bone health in childhood predictive of bone health in adulthood? Does bone health change over generations, and if yes, why? Is the well-described Flynn effect paralleled by a secular trend in growth parameters?

## Conclusion and Outlook

This article highlights the value of long-term longitudinal studies for aging research using the example of the ZLS. The article aims to foster the interdisciplinary discussion on how studies that were established to investigate child health and development may add to the understanding of adult health outcomes and aging-related processes in cognition, behavior, and other domains. The rich database of the ZLS includes data on physical, motor, cognitive, and social health and development and on the proximal and distal environment of individuals born with or without a risk for neurodevelopmental impairments and on parents and their children: a combination that makes these studies unique in the quest to study the importance of early life for later health and development.

In the future, the impact of the ZLS datasets may even be multiplied by collaborating with other researchers in combining datasets (see e.g., Friedman et al., 2014 for a call to integrate existing longitudinal studies to study the link between personality traits and health across the lifespan) and by the implementation of modern scientific technologies in future assessment waves (e.g., neuroimaging and genetic methods). Currently, the data of ZLS-1 participants is being aligned and compared with participants of the LifeStories study (see Lannen et al., 2021 for details). This will provide insights into how the early environment of an infant either placed in an infant care institution or growing up in a family setting affects health and development across the lifespan. Future comparison with further cohorts may help to disentangle the factors in early life that shape development into adulthood and across the lifespan.

A number of methodological and conceptual challenges remain to be addressed and solved to expand ZLS-Childhood with ZLS-Adulthood and to establish ZLS-Lifespan. However, this endeavor will make the ZLS invaluable for studying the importance of early life for health and development as individuals age.

## Dissemination

The findings derived from the ZLS datasets will be disseminated within the research community and beyond: Exchange and discourse with experts in the field of aging research and lifespan health development will be sought through contributions in scientific journals, choosing open access options whenever possible, and at scientific meetings. The study participants and the general public will be informed about research findings through media coverage, newsletters and information events. It is expected that the findings derived from the ZLS datasets will have a broad impact on society, as they have had in the past [e.g., with the long-term bestseller *Babyjahre* (Baby years) authored by Prof. emer. Remo H. Largo, the former Director of the Growth and Development Center of the University Children's Hospital and the former scientific leader of the ZLS and co-author of this article].

## Ethics Statement

The studies involving human participants were reviewed and approved by the Ethical committee of the Canton of Zurich, Switzerland (Basec-Nr. 2018-00686). The patients/participants provided their written informed consent to participate in this study.

## Author Contributions

FMW, a postdoctoral researcher and the current project leader of ZLS-Adulthood, drafted and edited the manuscript. JC, a senior scientist in the ZLS since 1993, examined numerous participants of the ZLS-3 cohort, has been responsible for archiving the ZLS-Childhood datasets and provided input to the mauscript. DAE, a PhD student in the ZLS, assesses participants for ZLS-Adulthood, prepares archived data for analyses, prepared data for several figures and provided input to the manuscript. GH, a research assistant in ZLS, locates and assesses participants for ZLS-Adulthood and provided input to the manuscript. BL, the co-director of the Child Development Center, University Children's Hospital Zurich, is responsible for the at-risk population in the ZLS and provided input to the manuscript. RHL was the former principal investigator of the ZLS (from 1974 to 2005), provided input to the manuscript and approved the initially submitted manuscript. Sadly, RHL died on November 11th 2020, shortly after manuscript submission. We dedicate this work to the memory of RHL. THK, a senior scientist in the ZLS, is responsible for the motor assessments in the ZLS, prepared several figures and photographs and provided input to the manuscript. OGJ, the current principal investigator of the ZLS (2005-current), drafted parts of the manuscript, provided input to all other sections, and edited the final version. All authors contributed to the article and approved the submitted version.

## Conflict of Interest

The authors declare that the research was conducted in the absence of any commercial or financial relationships that could be construed as a potential conflict of interest. The handling Editor declared a shared affiliation, though no other collaboration, with authors BL and OGJ at time of review.

## References

[B1] Aartsen M. J. Cheval B. Sieber S. Van der Linden B. W. Gabriel R. Courvoisier D. S.. (2019). Advantaged socioeconomic conditions in childhood are associated with higher cognitive functioning but stronger cognitive decline in older age. Proc. Natl. Acad. Sci. 116, 5478–5486. 10.1073/pnas.1807679116 30804194 PMC6431198

[B2] Abernethy B. (1988). Dual-task methodology and motor skills research: some applications and methodological constraints. J. Hum. Move. Stud. 14, 101–132.

[B3] Angermaier M. (1974). Psycholinguistischer Entwicklungstest: PET; Manual; Deutsche Bearbeitung des “Illinois Test of Psycholinguistic Abilities”. Weinheim.

[B4] Aronson L. (2020). Healthy aging across the stages of old age. Clin. Geriatr. Med. 36, 549–558. 10.1016/j.cger.2020.06.001 33010893

[B5] Aschenbrenner S. Tucha O. Lange K. W. (2000). Regensburger Wortflüssigkeits-Test (RWT) [Regensburger Verbal Fluency Test (RWT)]. Göttingen: Hogrefe Verlag.

[B6] Bartholomew K. Horowitz L. M. (1991). Attachment styles among young adults: a test of a four-category model. J. Pers. Soc. Psychol. 61:226. 10.1037/0022-3514.61.2.226 1920064

[B7] Batty G. D. Deary I. J. Gottfredson L. S. (2007). Premorbid (early life) IQ and later mortality risk: systematic review. Ann. Epidemiol. 17, 278–288. 10.1016/j.annepidem.2006.07.010 17174570

[B8] Bayley N. (1969). Bayley Scales of Infant Development: Birth to Two Years. New York, NY: Psychological Corporation.

[B9] Beierlein C. Kovaleva A. Kemper C. J. Rammstedt B. (2012). Ein Messinstrument zur Erfassung subjektiver Kompetenzerwartungen: Allgemeine Selbstwirksamkeit Kurzskala (ASKU). Available online at: https://psycharchives.zpid.de/handle/20.500.12034/431 (accessed December 18, 2020).

[B10] Belsky D. W. Caspi A. Houts R. Cohen H. J. Corcoran D. L. Danese A. (2015). Quantification of biological aging in young adults. PNAS. 112, E4104–E4110. 10.1073/pnas.1506264112 26150497 PMC4522793

[B11] Beurskens R. Bock O. (2012). Age-related deficits of dual-task walking: a review. Neural Plast. 2012:131608. 10.1155/2012/131608 22848845 PMC3403123

[B12] Birmingham K. Doyle A. (2009). Ethics and governance of a longitudinal birth cohort. Paediatr. Perinat. Epidemiol. 23, 39–50. 10.1111/j.1365-3016.2008.00995.x 19490444

[B13] Block J. (2008). The Q-Sort in Character Appraisal: Encoding Subjective Impressions of Persons Quantitatively. Washington, DC: American Psychological Association.

[B14] Block J. (1961). The Q-Sort Method in Personality Assessment and Psychiatric Research. Springfield, IL.

[B15] Bonhoeffer J. Jenni O. (2018). Das frühkindliche Spielverhalten–ein Spiegel der kognitiven Entwicklung. Pädiatrie up2date. 13, 303–321. 10.1055/s-0043-115419

[B16] Brandt J. Benedict R. H. (2001). Hopkins Verbal Learning Test–Revised: Professional Manual. Odessa, FL: Psychological Assessment Resources.

[B17] Brandt M. Deindl C. Hank K. (2012). Tracing the origins of successful aging: the role of childhood conditions and social inequality in explaining later life health. Soc. Sci. Med. 74, 1418–1425. 10.1016/j.socscimed.2012.01.004 22398143

[B18] Brunet O. Lézine I. (1951). Le Développement Psychologique de la Première Enfance. Paris: Presses Universitaires de France.

[B19] Bundesamt für Statistik (2014). Schweizerische Gesundheitsbefragung 2012 [Swiss Health Survey 2012]. Bern: Bundesamt für Statistik.

[B20] Cermakova P. Formanek T. Kagstrom A. Winkler P. (2018). Socioeconomic position in childhood and cognitive aging in Europe. Neurology. 91, e1602–e1610. 10.1212/WNL.0000000000006390 30258021 PMC6205684

[B21] Cheng B. Titterington D. M. (1994). Neural networks: a review from a statistical perspective. Stat. Sci. 9, 2–30. 10.1214/ss/1177010638

[B22] Chiu I. Piguet O. Diehl-Schmid J. Riedl L. Beck J. Leyhe T.. (2018). Facial emotion recognition performance differentiates between behavioral variant frontotemporal dementia and major depressive disorder. J Clin. Psychiatry. 79:16m11342. 10.4088/JCP.16m11342 29360290

[B23] Chopik W. J. Edelstein R. S. Grimm K. J. (2019). Longitudinal changes in attachment orientation over a 59-year period. J. Pers. Soc. Psychol. 116:598. 10.1037/pspp0000167 28771022

[B24] Chopik W. J. Grimm K. J. (2019). Longitudinal changes and historic differences in narcissism from adolescence to older adulthood. Psychol. Aging 34:1109. 10.1037/pag0000379 31804115

[B25] Clawson A. (1962). The Bender Visual Motor Gestalt Test for Children: A Manual. Los Angeles, CA: Western Psychological Services.

[B26] Concordat on Open Research Data (2016). Available online at: https://www.ukri.org/files/legacy/documents/concordatonopenresearchdata-pdf/ (accessed December 18, 2020)

[B27] Cooper R. Hardy R. Sayer A. A. Ben-Shlomo Y. Birnie K. Cooper C.. (2011). Age and gender differences in physical capability levels from mid-life onwards: the harmonisation and meta-analysis of data from eight UK cohort studies. PLoS ONE 6:e27899. 10.1371/journal.pone.0027899 22114723 PMC3218057

[B28] Crook T. H. Feher E. P. Larrabee G. J. (1992). Assessment of memory complaint in age-associated memory impairment: the MAC-Q. Int. Psychogeriatr. 4, 165–176. 10.1017/S1041610292000991 1477304

[B29] Daseking M. Petermann F. (2013). BRIEF-P Verhaltensinventar zur Beurteilung exekutiver Funktionen für das Kindergartenalter; deutschsprachige Adaptation des Behavior Rating Inventory of Executive Function-Preschool Version (BRIEF-P) von Gerard A. Gioia, Kimberly Andrews Espy und Peter K. Isquith; Manual. Bern: Huber.

[B30] Davis D. W. Turkheimer E. Finkel D. Beam C. Ryan L. (2019). The Louisville twin study: past, present and future. Twin Res. Hum. Genet. 22, 735–740. 10.1017/thg.2019.37 31362801

[B31] Davis M. H. (1983). Measuring individual differences in empathy: evidence for a multidimensional approach. J. Pers. Soc. Psychol. 44:113 10.1037/0022-3514.44.1.113

[B32] Deary I. J. Gow A. J. Pattie A. Starr J. M. (2012). Cohort profile: the Lothian birth cohorts of 1921 and 1936. Int. J. Epidemiol. 41, 1576–1584. 10.1093/ije/dyr197 22253310

[B33] Diggle P. (2002). Analysis of longitudinal data. Oxford: Oxford University Press.

[B34] Dimitropoulos A. Molinari L. Etter K. Torresani T. Lang-Muritano M. Jenni O. G.. (2009). Children with congenital hypothyroidism: long-term intellectual outcome after early high-dose treatment. Pediatr. Res. 65, 242–248. 10.1203/PDR.0b013e31818d2030 18787501

[B35] Elliott K. C. Cheruvelil K. S. Montgomery G. M. Soranno P. A. (2016). Conceptions of good science in our data-rich world. Bioscience 66, 880–889. 10.1093/biosci/biw115 29599533 PMC5862324

[B36] Falkner F. (1960). Child Development: An International Method of Study. New York, NY: S. Karger.

[B37] Federal Statistical Office (2017). Census 2019. Available online at: https://www.bfs.admin.ch/bfs/en/home/basics/census.html

[B38] Franke K. Gaser C. Roseboom T. J. Schwab M. de Rooij S. R. (2018). Premature brain aging in humans exposed to maternal nutrient restriction during early gestation. Neuroimage 173, 460–471. 10.1016/j.neuroimage.2017.10.047 29074280

[B39] Franke G. H. (2017). Mini-SCL - Mini-Symptom-Checklist. 1. Auflage ed. Göttingen: Hogrefe.

[B40] Frey B. B. (2018). The SAGE Encyclopedia of Educational Research, Measurement, and Evaluation. Thousand Oaks, CA: Sage Publications.

[B41] Friedman H. Martin L. R. (2011). The Longevity Project: Surprising Discoveries for Health and Long Life From the Landmark Eight Decade Study. New York, NY: Hay House, Inc.

[B42] Friedman H. S. Kern M. L. Hampson S. E. Duckworth A. L. (2014). A new life-span approach to conscientiousness and health: combining the pieces of the causal puzzle. Dev. Psychol. 50:1377. 10.1037/a0030373 23088747 PMC3651756

[B43] Friedman H. S. Kern M. L. Reynolds C. A. (2010). Personality and health, subjective well-being, and longevity. J. Pers. 78, 179–216. 10.1111/j.1467-6494.2009.00613.x 20433617

[B44] Fydrich T. Sommer G. Brähler E. (2007). F-SOZU: Fragebogen zur sozialen Unterstützung. Göttingen: Hogrefe.

[B45] Gale C. R. Booth T. Starr J. M. Deary I. J. (2016). Intelligence and socioeconomic position in childhood in relation to frailty and cumulative allostatic load in later life: the Lothian Birth Cohort 1936. J. Epidemiol. Community Health 70, 576–582. 10.1136/jech-2015-205789 26700299 PMC4820036

[B46] Garnefski N. Kraaij V. Spinhoven P. (2001). Negative life events, cognitive emotion regulation and emotional problems. Pers. Individ. Diff. 30, 1311–1327. 10.1016/S0191-8869(00)00113-616600359

[B47] Gasser T. Köhler W. Müller H. Kneip A. Largo R. Molinari L.. (1984). Velocity and acceleration of height growth using kernel estimation. Ann. Hum. Biol. 11, 397–411. 10.1080/03014468400007311 6486712

[B48] Gierveld J. D. J. Tilburg T. V. (2006). A 6-item scale for overall, emotional, and social loneliness: confirmatory tests on survey data. Res. Aging 28, 582–598. 10.1177/016402750628972318365511

[B49] Gilsoul J. Simon J. Hogge M. Collette F. (2019). Do attentional capacities and processing speed mediate the effect of age on executive functioning? Aging Neuropsychol. Cogn. 26, 282–317. 10.1080/13825585.2018.1432746 29405831

[B50] Glaesmer H. Grande G. Braehler E. Roth M. (2011). The German version of the satisfaction with life scale (SWLS). Eur. J. Psychol. Assess. 27, 127–132. 10.1027/1015-5759/a000058

[B51] Golding J. (2009a). Measuring outcomes in a longitudinal birth cohort. Paediatr. Perinat. Epidemiol. 23, 185–200. 10.1111/j.1365-3016.2009.01016.x 19490456

[B52] Golding J. (2009b). Who should be studied and when in a longitudinal birth cohort? Paediatr. Perinat. Epidemiol. 23, 15–22. 10.1111/j.1365-3016.2008.00998.x 19490441

[B53] Golding J. (2010). Are findings from large longitudinal studies of child health and development useful or just of interest? Paediatr. Child Health 20, 163–166. 10.1016/j.paed.2009.12.003

[B54] Goldstein S. Naglieri J. A. Princiotta D. Otero T. M. (2014). Introduction: A History of Executive Functioning as a Theoretical and Clinical Construct. Handbook of Executive Functioning. Springer 3–12.

[B55] Goodenough F. L. (1926). Measurement of Intelligence by Drawings. New York, NY: Harcourt Brace.

[B56] Griffiths R. (1954). The Abilities of Babies: A Study in Mental Measurement. New York, NY: McGraw-Hill.

[B57] Grimm K. J. McArdle J. Widaman K. F. (2011). Family-level variance in verbal ability change in the intergenerational studies in Secondary Data Analysis: An Introduction for Psychologists, eds Trzesniewski K. H. Donnellan M. B. Lucas R. E. (Washington, DC: American Psychological Association), 209–229.

[B58] Grosse Holtforth M. Grawe K. (2003). Der Inkongruenzfragebogen (INK). Zeitschrift klinische Psychol. Psychother. 32, 315–323. 10.1026/0084-5345.32.4.315

[B59] Harris D.B. (1963). Children's Drawings as Measures of Intellectual Maturity. New York, NY: Harcourt, Brace & World.

[B60] Harris P. A. Taylor R. Minor B. L. Elliott V. Fernandez M. O'Neal L.. (2019). The REDCap consortium: building an international community of software platform partners. J. Biomed. Inform. 95:103208. 10.1016/j.jbi.2019.103208 31078660 PMC7254481

[B61] Harris P. A. Taylor R. Thielke R. Payne J. Gonzalez N. Conde J. G. (2009). Research electronic data capture (REDCap)—a metadata-driven methodology and workflow process for providing translational research informatics support. J. Biomed. Inform. 42, 377–381. 10.1016/j.jbi.2008.08.010 18929686 PMC2700030

[B62] Heim A. (1970). Manual for the AH4 Group Test of General Intelligence. Windsor: NFER.

[B63] Hinz A. Glaesmer H. Brähler E. Löffler M. Engel C. Enzenbach C.. (2017). Sleep quality in the general population: psychometric properties of the Pittsburgh sleep quality index, derived from a German community sample of 9284 people. Sleep Med. 30, 57–63. 10.1016/j.sleep.2016.03.008 28215264

[B64] Hofer S. M. Piccinin A. M. (2009). Integrative data analysis through coordination of measurement and analysis protocol across independent longitudinal studies. Psychol. Methods 14:150. 10.1037/a0015566 19485626 PMC2773828

[B65] Horvath S. Raj K. (2018). DNA methylation-based biomarkers and the epigenetic clock theory of ageing. Nat. Rev. Genet. 19:371. 10.1038/s41576-018-0004-3 29643443

[B66] Iglowstein I. Jenni O. G. Molinari L. Largo R. H. (2003). Sleep duration from infancy to adolescence: reference values and generational trends. Pediatrics 111, 302–307. 10.1542/peds.111.2.302 12563055

[B67] Jaddoe V. W. Mackenbach J. P. Moll H. A. Steegers E. A. Tiemeier H. Verhulst F. C.. (2006). The generation R study: design and cohort profile. Eur. J. Epidemiol. 21:475. 10.1007/s10654-006-9022-0 16826450

[B68] Jenni O. (2013). Wie Kinder die Welt abbilden–und was man daraus folgern kann. Pädiatr. up2date. 8, 227–253. 10.1055/s-0032-1326475

[B69] Jenni O. G. Chaouch A. Caflisch J. Rousson V. (2013). Infant motor milestones: poor predictive value for outcome of healthy children. Acta Paediatr. 102, e181–e184. 10.1111/apa.12129 23289493

[B70] Jenni O. G. Chaouch A. Locatelli I. Thoeni I. Diezi M. Werner H.. (2011). Intra-individual stability of neuromotor tasks from 6 to 18 years: a longitudinal study. Hum. Move. Sci. 30, 1272–1282. 10.1016/j.humov.2010.12.002 21813200

[B71] Jenni O. G. Molinari L. Caflisch J. A. Largo R. H. (2007). Sleep duration from ages 1 to 10 years: variability and stability in comparison with growth. Pediatrics 120, e769–e776. 10.1542/peds.2006-3300 17908734

[B72] Jones C. (2010). Archival data: advantages and disadvantages for research in psychology. Soc. Pers. Psychol. Compass 4, 1008–1017. 10.1111/j.1751-9004.2010.00317.x

[B73] Jones C. J. Meredith W. (1996). Patterns of personality change across the life span. Psychol. Aging 11:57 10.1037/0882-7974.11.1.57 8726370

[B74] Jones C. J. Peskin H. (2010). Psychological health from the teens to the 80s: multiple developmental trajectories. J. Adult Dev. 17:20 10.1007/s10804-009-9075-x

[B75] Jones R. (2009). The importance of biological samples in longitudinal birth cohort studies. Paediatr. Perinat. Epidemiol. 23, 93–102. 10.1111/j.1365-3016.2008.00999.x 19490449

[B76] Kakebeeke T. H. Knaier E. Chaouch A. Caflisch J. Rousson V. Largo R. H.. (2018). Neuromotor development in children. Part 4: new norms from 3 to 18 years. Dev. Med. Child Neurol. 60, 810–819 10.1111/dmcn.13793 29732550

[B77] Kell D. B. Oliver S. G. (2004). Here is the evidence, now what is the hypothesis? The complementary roles of inductive and hypothesis-driven science in the post-genomic era. Bioessays 26, 99–105. 10.1002/bies.10385 14696046

[B78] Kern M. L. Della Porta S. S. Friedman H. S. (2014). Lifelong pathways to longevity: personality, relationships, flourishing, and health. J. Pers. 82, 472–484. 10.1111/jopy.12062 23927423

[B79] Kliem S. Job A.-K. Kröger C. Bodenmann G. Stöbel-Richter Y. Hahlweg K. (2012). Entwicklung und Normierung einer Kurzform des Partnerschaftsfragebogens (PFB-K) an einer repräsentativen deutschen Stichprobe. Zeitschrift Klinische Psychol. Psychother. 41, 81–89. 10.1026/1616-3443/a000135

[B80] Körner A. Geyer M. Roth M. Drapeau M. Schmutzer G. Albani C.. (2008). Persönlichkeitsdiagnostik mit dem neo-fünf-faktoren-inventar: Die 30-item-kurzversion (neo-ffi-30). PPmP Psychother. Psychosomatik Medizinische Psychol. 58, 238–245. 10.1055/s-2007-986199 17899495

[B81] Kubinger K. D. Wurst E. (1985). Adaptives Intelligenz-Diagnostikum: AID. Weinheim: Beltz. 2750245

[B82] Kuh D. (2016). From paediatrics to geriatrics: a life course perspective on the MRC national survey of health and development. Eur. J. Epidemiol. 31, 1069–1079. 10.1007/s10654-016-0214-y 28004211 PMC5206253

[B83] Kuh D. Pierce M. Adams J. Deanfield J. Ekelund U. Friberg P.. (2011). Cohort profile: updating the cohort profile for the MRC national survey of health and development: a new clinic-based data collection for ageing research. Int. J. Epidemiol. 40:e1–e9. 10.1093/ije/dyq231 21345808 PMC3043283

[B84] Kuh D. Wong A. Shah I. Moore A. Popham M. Curran P.. (2016). The MRC national survey of health and development reaches age 70: maintaining participation at older ages in a birth cohort study. Eur. J. Epidemiol. 31, 1135–1147. 10.1007/s10654-016-0217-8 27995394 PMC5206260

[B84a] Lannen P. Sand H. Sticca F. Gallego I. R. Bombach C. Simoni H. (2021). Development and health of adults formerly placed in infant care institutions - study protocol of the life stories project. Front. Hum. Neurosci. 10.3389/fnhum.2020.611691 PMC7854920 33551778

[B85] Largo R. Gasser T. Prader A. Stuetzle W. Huber P. (1978). Analysis of the adolescent growth spurt using smoothing spline functions. Ann. Hum. Biol. 5, 421–434. 10.1080/03014467800003071 727700

[B86] Largo R. Molinari L. Pinto L. C. Weber M. Due G. (1986). Language development of term and preterm children during the first five years of life. Dev. Med. Child Neurol. 28, 333–350. 10.1111/j.1469-8749.1986.tb03882.x 3721077

[B87] Largo R. H. Caflisch J. A. Hug F. Muggli K. Molnar A. A. Molinari L.. (2001a). Neuromotor development from 5 to 18 years. Part 1: timed performance. Dev. Med. Child Neurol. 43, 436–443. 10.1111/j.1469-8749.2001.tb00739.x 11463173

[B88] Largo R. H. Caflisch J. A. Hug F. Muggli K. Molnar A. A. Molinari L. (2001b). Neuromotor development from 5 to 18 years. Part 2: associated movements. Dev. Med. Child Neurol. 43, 444–453. 10.1111/j.1469-8749.2001.tb00740.x 11463174

[B89] Largo R. H. Molinari L. Siebenthal K. Wolfensberger U. (1996). Does a profound change in toilet-training affect development of bowel and bladder control? Dev. Med. Child Neurol. 38, 1106–1116. 10.1111/j.1469-8749.1996.tb15074.x 8973296

[B90] Launes J. Hokkanen L. Laasonen M. Tuulio-Henriksson A. Virta M. Lipsanen J.. (2014). Attrition in a 30-year follow-up of a perinatal birth risk cohort: factors change with age. PeerJ 2:e480. 10.7717/peerj.480 25071998 PMC4103077

[B91] Lienhard K. Schneider D. Maffiuletti N. A. (2013). Validity of the optogait photoelectric system for the assessment of spatiotemporal gait parameters. Med. Eng. Phys. 35, 500–504. 10.1016/j.medengphy.2012.06.015 22818403

[B92] Loch N. Hiller W. Witthöft M. (2011). Der cognitive emotion regulation questionnaire (CERQ). Zeitschrift für Klinische Psychologie und Psychotherapie. 40, 94–106. 10.1026/1616-3443/a000079

[B93] Lückert H.-R. (1957). Stanford Intelligenz-Test: Handanweisung;[SIT]. Verlag f. Göttingen: Psychologie, Hogrefe.

[B94] Magoc T. Magoc D. (2011). Neural network to identify individuals at health risk. Int. J. Artif. Intel. Appl. 2, 104–114. 10.5121/ijaia.2011.2208

[B95] Matsumuro M. Miwa K. (2019). Model for data analysis process and its relationship to the hypothesis-driven and data-driven research approaches, in International Conference on Intelligent Tutoring Systems (Springer).

[B96] McArdle J. J. Grimm K. J. Hamagami F. Bowles R. P. Meredith W. (2009). Modeling life-span growth curves of cognition using longitudinal data with multiple samples and changing scales of measurement. Psychol. Methods 14:126. 10.1037/a0015857 19485625 PMC2831479

[B97] McCrory C. Dooley C. Layte R. Kenny R. A. (2015). The lasting legacy of childhood adversity for disease risk in later life. Health Psychol. 34:687 10.1037/hea0000147 25150540

[B98] Moffitt T. E. Arseneault L. Belsky D. Dickson N. Hancox R. J. Harrington H.. (2011). A gradient of childhood self-control predicts health, wealth, and public safety. Proc. Natl. Acad. Sci. 108, 2693–2698. 10.1073/pnas.1010076108 21262822 PMC3041102

[B99] Moffitt T. E. Belsky D. W. Danese A. Poulton R. Caspi A. (2017). The longitudinal study of aging in human young adults: knowledge gaps and research agenda. J. Gerontol. A Biol. Sci. Med. Sci. 72, 210–215. 10.1093/gerona/glw191 28087676 PMC5233916

[B100] Morfeld M. Kirchberger I. Bullinger M. (2011). Fragebogen zum Gesundheitszustand (SF-36). Bern: Hogrefe.

[B101] Mroczek D. K. Pitzer L. Miller L. Turiano N. Fingerman K. (2011). The use of secondary data in adult development and aging research, in Secondary Data Analysis: An Introduction for Psychologists, eds Trzesniewski K. H. Donnellan M. B. Lucas R. E. (Washington, DC: American Psychological Association), 121–132. 10.1037/12350-007

[B102] Naef N. Wehrle F. Rousson V. Latal B. (2019). Cohort and individual neurodevelopmental stability between 1 and 6 years of age in children with congenital heart disease. J. Pediatr. 215, 83–89.e2. 10.1016/j.jpeds.2019.08.036 31563274

[B103] Nasreddine Z. S. Phillips N. A. Bédirian V. Charbonneau S. Whitehead V. Collin I. (2005). The Montreal Cognitive Assessment, MoCA: a brief screening tool for mild cognitive impairment. J. Am. Geriatr. Soc. 53, 695–699. 10.1111/j.1532-5415.2005.53221.x 15817019

[B104] Nettelbeck T. Burns N. R. (2010). Processing speed, working memory and reasoning ability from childhood to old age. Pers. Individ. Diff. 48, 379–384. 10.1016/j.paid.2009.10.032

[B105] Neumann E. (2002). Die Paarbeziehung Erwachsener und Erinnerungen an die Eltern-Kind-Beziehung: eine Untersuchung zur Kontinuität von Bindung. Zeitschrift Familienforschung 14, 234–256. Available online at: https://nbn-resolving.org/urn:nbn:de:0168-ssoar-282823

[B106] Nishizawa T. Morita A. Fujiwara T. Kondo K. (2019). Association between childhood socioeconomic status and subjective memory complaints among older adults: results from the Japan gerontological evaluation study 2010. Int. Psychogeriatr. 31, 1699–1707. 10.1017/S1041610219000814 31317850

[B107] Oh J. Chopik W. J. Konrath S. Grimm K. J. (2020). Longitudinal changes in empathy across the life span in six samples of human development. Soc. Psychol. Pers. Sci. 11, 244–253. 10.1177/1948550619849429

[B108] Paulus C. (2011). Der Saarbrücker Persönlichkeitsfragebogen zur Messung von Empathie (SPF-IRI). Available online at: http://bildungswissenschaften.uni-saarland.de/personal/paulus/homepage/empathie.html (accessed December 18, 2020)

[B109] Peskin H. Jones C. (2015). Early-and late-adolescent predictors of psychological health in adulthood: results from the intergenerational studies. J. Adult Dev. 22, 230–238. 10.1007/s10804-015-9214-5

[B110] Petermann F. Petermann U. (2012). Wechsler Adult Intelligence Scale–Fourth Edition. Deutschsprachige Adaptation der WAIS-IV von D. Wechsler. Frankfurt/Main: Pearson Assessment.

[B111] Pienta A. M. Lyle J. (2018). Retirement in the 1950s: rebuilding a longitudinal research database. IASSIST Q. 42, 1–9. 10.29173/iq19 30853751 PMC6404736

[B112] Poulton R. Moffitt T. E. Silva P. A. (2015). The Dunedin multidisciplinary health and development study: overview of the first 40 years, with an eye to the future. Soc. Psychiatry Psychiatr. Epidemiol. 50, 679–693. 10.1007/s00127-015-1048-8 25835958 PMC4412685

[B113] Prader A. Largo R. H. Molinari L. Issler C. (1989). Physical growth of Swiss children from birth to 20 years of age. First Zurich longitudinal study of growth and development. Helvetica Paediatr. Acta Suppl. 52, 1–125. 2737921

[B114] Raven J. C. (1947). Progressive Matrices 1947, Sets A, AB, B [and] Guide to Using Progressive Matrices (1947), Sets A, AB, B. London: Lewis.

[B115] Raven J. C. (1956). Manuel D'Instruction du Matrix (Matrices Progressives, 1947). Paris: Editions Scientifiques et Psychotechniques.

[B116] Regard M. Strauss E. Knapp P. (1982). Children's production on verbal and non-verbal fluency tasks. Percept. Motor Skills 55, 839–844. 10.2466/pms.1982.55.3.839 7162920

[B117] Rey A. (1941). L'examen psychologique dans les cas d'encéphalopathie traumatique. (Les problems.). Arch. Psychol. 28, 215–285. 16987634

[B118] Roche A. F. (1992). Growth, Maturation, and Body Composition: The Fels Longitudinal Study 1929-1991. Cambridge: Cambridge University Press.

[B119] Roseboom T. de Rooij S. Painter R. (2006). The Dutch famine and its long-term consequences for adult health. Early Hum. Dev. 82, 485–491. 10.1016/j.earlhumdev.2006.07.001 16876341

[B120] Rosenzweig S. Fleming E. E. Rosenzweig L. Duhm E. Hansen J. (1948). Der Rosenzweig P-F Test: The Picture Frustration Study von Saul Rozenzweig. Göttingen: Verlag fur Psychologie Hogrefe.

[B121] Rousson V. Gasser T. (2004). Simple component analysis. J. R. Stat. Soc. Series C. 53, 539–555. 10.1111/j.1467-9876.2004.05359.x

[B122] Ryff C. D. Keyes C. L. M. (1995). The structure of psychological well-being revisited. J. Pers. Soc. Psychol. 69:719. 10.1037/0022-3514.69.4.719 7473027

[B123] Sabo R. Wang A. Deng Y. Sabo C. Sun S. (2017). Relationships between childhood growth parameters and adult blood pressure: the Fels Longitudinal Study. J. Dev. Orig. Health Dis. 8, 113–122. 10.1017/S2040174416000520 27628681

[B124] Salthouse T. A. (1996). The processing-speed theory of adult age differences in cognition. Psychol. Rev. 103:403. 10.1037/0033-295X.103.3.403 8759042

[B125] Sanders A. E. (2016). Shifting the focus of aging research into earlier decades of life. Oral Dis. 22, 166–168. 10.1111/odi.12431 26713862

[B126] Schaefer J. D. Caspi A. Belsky D. W. Harrington H. Houts R. Israel S.. (2015). Early-life intelligence predicts midlife biological age. J. Gerontol. B Psychol. Sci. Soc. Sci. 71, 968–977. 10.1093/geronb/gbv035 26014827 PMC5067943

[B127] Schmidt S. Muehlan H. Brähler E. (2016). AAS-R-Revised Adult Attachment Scale: Deutsche Version. Göttingen: Hogrefe.

[B128] Schuhmacher J. Wilz G. Gunzelmann T. Brähler E. (2000). Die sense of coherence scale von Antonovsky. Teststatistische Überprüfung in einer representativen Bevölkerungsstichprobe und Konstruktion einer Kurzskala. PPmP Psychother Psychosom med Psychol. 50, 472–482. 10.1055/s-2000-9207 11199111

[B129] Siedler T. Schupp J. Wagner G. G. (2011). Innovative methods within the context of secondary data: examples from household panel surveys, in Secondary Data Analysis: An Introduction for Psychologists, eds Trzesniewski K. H. Donnellan M. B. Lucas R. E. (Washington, DC: American Psychological Association), 103–118.

[B130] Snijders-Oomen N. (1977). Snijders-Oomen nicht verbale Intelligenztestreihe: SON 2 1/2-7. Groningen: Wolters-Noordhoff.

[B131] Stafford M. Gale C. R. Mishra G. Richards M. Black S. Kuh D. L. (2015). Childhood environment and mental wellbeing at age 60-64 years: prospective evidence from the MRC national survey of health and development. PLoS ONE 10:e0126683. 10.1371/journal.pone.0126683 26030929 PMC4451971

[B132] Staudinger U. M. Fleeson W. Baltes P. B. (1999). Predictors of subjective physical health and global well-being: similarities and differences between the United States and Germany. J. Pers. Soc. Psychol. 76:305 10.1037/0022-3514.76.2.305

[B133] Stevenson A. J. McCartney D. L. Hillary R. F. Redmond P. Taylor A. M. Zhang Q.. (2019). Childhood intelligence attenuates the association between biological ageing and health outcomes in later life. Trans. Psychiatry 9, 1–8. 10.1038/s41398-019-0657-5 31780646 PMC6883059

[B134] Steyer R. Schwenkmezger P. Notz P. Eid M. (1997). Der mehrdimensionale Befindlichkeitsfragebogen (MDBF). [The Multidimensional Mood Questionnaire (MMQ)]. Göttingen: Hogrefe.

[B135] Strauss E. Sherman E. M. S. Spreen O. (2006). A Compendium of Neuropsychological Tests: Administration, Norms, and Commentary, 3rd Edn. New York, NY: Oxford University Press.

[B136] Sun S. S. Liang R. Huang T. T.-K. Daniels S. R. Arslanian S. Liu K.. (2008). Childhood obesity predicts adult metabolic syndrome: the Fels Longitudinal Study. J. Pediatr. 152, 191–200.e1. 10.1016/j.jpeds.2007.07.055 18206688 PMC3988700

[B137] Tanner J. (1962). Growth at Adolescence. Oxford: Blackwell Scientific Publications.

[B138] Tanner J. M. (1981). A History of the Study of Human growth. Cambridge: Cambridge University Press.

[B139] Tanner J. M. (1998). A Brief History of the Study of Human Growth. Cambridge: The Cambridge Encyclopedia of Human Growth and Development University Press 2–7.

[B140] Taylor A. M. Pattie A. Deary I. J. (2018). Cohort profile update: the Lothian birth cohorts of 1921 and 1936. Int. J. Epidemiol. 47, 1042–1042r. 10.1093/ije/dyy022 29546429 PMC6124629

[B141] Terman L. M. Merrill M. A. (1937). Measuring Intelligence: A Guide to the Administration of the New Revised Stanford-Binet tests of Intelligence. Boston: Houghton Mifflin.

[B142] Terman L. M. Merrill M. A. (1960). Stanford-Binet Intelligence scale: Manual for the Third Revision, Form L-M. Boston: Houghton Mifflin.

[B143] Terman L. M. (1916). The Measurement of Intelligence: An Explanation of and a Complete Guide for the Use of the Stanford Revision and Extension of the Binet-Simon Intelligence Scale. Boston: Houghton Mifflin.

[B144] Terman L. M. Merrill M. A. Lückert H.-R. (1965). Stanford-Binet Intelligenz-Test, SIT. Handanweisung: Verlag f. Psychologie Hogrefe.

[B145] Tewes U. Titze I. (1983). Untersuchungen zur Anwendung des HAWIK in der klinischen und sonderpdagogischen Diagnostik [Studies on the application of HAWIK in clinical and special educational diagnosis]. Zeitschrift Differentielle Diagnostische Psychol. 4, 179-201.

[B146] The Federal Assembly of the Swiss Confederation (2014). Federal Act on Research Involving Human Beings. Available online at: https://www.admin.ch/opc/en/classified-compilation/20061313/index.html (accessed December 18, 2020)

[B147] Thodberg H. H. Jenni O. G. Caflisch J. Ranke M. B. Martin D. D. (2009). Prediction of adult height based on automated determination of bone age. J. Clin. Endocrinol. Metabol. 94, 4868–4874. 10.1210/jc.2009-1429 19926715

[B148] Unterrainer J. M. Rahm B. Kaller C. P. Wild P. S. Münzel T. Blettner M.. (2019). Assessing planning ability across the adult life span in a large population-representative sample: reliability estimates and normative data for the tower of London (TOL-F) task. J. Int. Neuropsychol. Soc. 25, 520–529. 10.1017/S1355617718001248 30696511 PMC6669988

[B149] Van den Broek T. Fleischmann M. (2019). Prenatal famine exposure and mental health in later midlife. Aging Ment. Health 23, 166–170. 10.1080/13607863.2017.1402293 29125320

[B150] Veale J. F. (2014). Edinburgh handedness inventory–short form: a revised version based on confirmatory factor analysis. Laterality 19, 164–177. 10.1080/1357650X.2013.783045 23659650

[B151] Verbeke G. Molenberghs G. (2000). Linear Mixed Models for Longitudinal Data. New York, NY: Springer.

[B152] Voorpostel M. Tillmann R. Lebert F. Kuhn U. Lipps O. Ryser V.-A. (2016). Swiss Household Panel User Guide (1999–2015). Lausanne: FORS.

[B153] Wadsworth M. Kuh D. Richards M. Hardy R. (2006). Cohort profile: the 1946 national birth cohort (MRC national survey of health and development). Int. J. Epidemiol. 35, 49–54. 10.1093/ije/dyi201 16204333

[B154] Weiss D. Job V. Mathias M. Grah S. Freund A. M. (2016). The end is (not) near: aging, essentialism, and future time perspective. Dev. Psychol. 52:996. 10.1037/dev0000115 27228453

[B155] Williamson W. Leroi I. (2019). Thinking about dementia: is childhood too early? Int. Psychogeriatr. 31, 1689–1690. 10.1017/S1041610219001157 31856931

[B156] Willich O. Friese H. (1994). Der Hamburg-Wechsler-Intelligenztest für Kinder revision 1983 (HAWIK-R). Diagnostica. 40, 172–189.

[B157] Wirtz M. A. Morfeld M. Glaesmer H. Brähler E. (2018). Konfirmatorische Prüfung der Skalenstruktur des SF-12 Version 2.0 in einer deutschen bevölkerungs-repräsentativen Stichprobe [Confirmatory analysis of the SF-12 version 2.0 scale structure in a representative German sample.]. Diagnostica. 64, 84–96. 10.1026/0012-1924/a000194

[B158] World Health Organization (1948). Preamble to the Constitution of WHO as adopted by the International Health Conference, New York, 19 June−22 July 1946; Signed on 22 July 1946 by the Representatives of 61 States (Official Records of WHO, no. 2. p. 100.) and Entered Into Force on 7 April 1948. Geneva: WHO.

[B159] World Health Organization (2016). Global Strategy and Action Plan on Ageing and Health (2016–2020). Geneva: World Health Organization.

[B160] Young A. F. Powers J. R. Bell S. L. (2006). Attrition in longitudinal studies: who do you lose? Aust. N. Zeal. J. Public Health 30, 353–361. 10.1111/j.1467-842X.2006.tb00849.x 16956166

[B161] Zimmermann P. Fimm B. (2009). Testbatterie zur Aufmerksamkeitsprüfung-Version 2.2:(TAP);[Handbuch]. Herzogenrath: Psytest.

