# Supplementary material for: The Importance of Childhood for Adult Health and Development—Study Protocol of the Zurich Longitudinal Studies
Source: Front Hum Neurosci. 2021 Jan 28;14:612453. doi: 10.3389/fnhum.2020.612453 (PMC7901945; doi:10.3389/fnhum.2020.612453)
Supplement: Supplementary file 1 [file Data_Sheet_1.pdf]

Supplementary Material

Supplementary 1. English translation of ZLS-Childhood record form to assess anthropometric measures and questionnaire to assess sleep quantity and quality (extract).

SOMATIC MEASUREMENTS

Case no.:	……………………………………………………...	1-3
Sex:	female (8) – male (9)	4
Recruitment Group:	1-114 (1) – 115-218 (2) – 219-306 (3)<br>307- 418 (4)	5
Card ID:		6
Examination:	none (0) – at correct time (9)<br>at incorrect time (5)	7
Examiner:	…………………………………………………	8
Age:	birth (80) – weeks: 4 (70) – 13 (60) – 26 (50)<br>39 (40) – years : 1 (39) – 1½ (20)<br>2 – 18 (02- 18)	9-10

MEASUREMENTS

Weight		kg/100	11-14
Supine length		mm	15-18
Crow-rump length		mm	19-22
Standing height		mm	23-26
Sitting height		mm	27-30
Chest circumference		mm	31-33
Head circumference		mm	34-36
Upper arm circumference		mm	37-39
Calf circumference		mm	40-42
Knee diameter		mm	43-45
Elbow diameter		mm	46-48
Pelvic diameter		mm	49-51
Shoulder width		mm	52-54
Skinfold	Biceps	mm/10	55-57
	Triceps	mm/10	58-60
	Subscapular	mm/10	61-63
	subiliacal	mm/10	64-66
empty			67-69

Skull diameter biparietal	mm		70-72
fronto-occipital	mm		73-75
Arm length	cm		76-77
Month of birth chronological:	Jan.=1 – Dec.=12		78

Impression of examiner regarding health status since last visit (79)

healthy (6) – not healthy with impact on development (7) –
potential impact (8) – likely without impact (9)

Current health status (80)

Very healthy (11) – healthy (9) –
sick (6) – somewhat sick (7) – recovering (8)
likely no meaning (9)

SLEEP

How has he been sleeping? 47

not well (6) – fairly well (7) – very well (8) – extr. well (9)

Complaints (since last visit) 48

other or remarks or doubtful (12)

poor day sleeper (11) – no complaints (0)

wakeful evenings (1) – resists going to sleep (2)

Wakeful at night with crying (3) – wakeful at night without crying (4)

restless (5) – night terrors (6)

bad dreams (7) - talks in sleep (8)

sleep walking (9)

Sleepingtimes

Day: nil (0) – (one nap) (1) 49

(two naps) (2) – (three naps or more) (3)

Up to 1 hr (total) (5) – up to 2 hrs (6)

up to 3 hrs (7) – up to 4 hrs (8) – more 4 hrs (9)

Night: less. sp. (1)- 7 hrs (2) – 8 hrs (3) 50

9 hrs (4) – 10 hrs (5) – 11 hrs (6) – 12 hrs (7) – 13 hrs (8) – more (9)

Regularity of these sleeping times:

Day: (no day sleep) (0)- very irregular (7) 51

some variation (8) – very regular (9)

Night: very irregular (7) – some variation (8) 52

very regular (9)

Usual bedtime: 53

17 and earlier (1) – 18 (2) – 18.30 (3) – 19 (4)

19.30 (5) – 20 (6) – 20.30 (7) – 21 (8) 22 and later (9)

Usual waking time: 54

4 and earlier (1) – 5 (2) – 5.30 (3) 6 (4)

6.30 (5) – 7 (6) – 7.30 (7) 8 (8) – 9 and later (9)

Have you had any trouble getting him off to sleep or has he been waking in the evening (before parent's bed time)?

a)resists preparation 55

never (0) – never now, but yes since last visit (6)

Now, yes, rarely (7) – sometimes (8) – usually (9)

b)waking 56

Demands attention in evening: 57

never (0) – never now, but yes since last visit (6)

now, yes, rarely (7) sometimes (8) – usually (9)

Demands by: (no demand) (0) – crying (6) – calling (7) 58

coming out (8) – other (9)

Supplementary 2. **Questions to assess the impact of the Covid-19 pandemic on the lives of the study participants (English translation; questions added in June 2020).**

If you are (still) working:

How much has the COVID-19 pandemic changed your work situation (e.g. home office, overtime, loss of employment …)?	☐ 1 not at all<br>☐ 2<br>☐ 3<br>☐ 4<br>☐ 5 very strongly
How stressful is this for you?	☐ 1 not at all stressful<br>☐ 2<br>☐ 3<br>☐ 4<br>☐ 5 very stressful
How much positive effect does this cause?	☐ 1 none<br>☐ 2<br>☐ 3<br>☐ 4<br>☐ 5 very much

Financial situation:

How much has the COVID-19 pandemic changed your financial situation?	☐ 1 not at all<br>☐ 2<br>☐ 3<br>☐ 4<br>☐ 5 very strongly
How stressful is this for you?	☐ 1 not at all stressful<br>☐ 2<br>☐ 3<br>☐ 4<br>☐ 5 very stressful
How much positive effect does this cause?	☐ 1 none<br>☐ 2<br>☐ 3<br>☐ 4<br>☐ 5 very much

If you live with other people:

How much has the COVID-19 pandemic changed your <u>family environment</u>?

- ☐ 1 not at all
- ☐ 2
- ☐ 3
- ☐ 4
- ☐ 5 very strongly

How stressful is this for you?

- ☐ 1 not at all stressful
- ☐ 2
- ☐ 3
- ☐ 4
- ☐ 5 very stressful

How much positive effect does this cause?

- ☐ 1 none
- ☐ 2
- ☐ 3
- ☐ 4
- ☐ 5 very much

If you are in a relationship:

How much has the COVID-19 pandemic changed your <u>relationship with spouse</u>?

- ☐ 1 not at all
- ☐ 2
- ☐ 3
- ☐ 4
- ☐ 5 very strongly

How stressful is this for you?

- ☐ 1 not at all stressful
- ☐ 2
- ☐ 3
- ☐ 4
- ☐ 5 very stressful

How much positive effect does this cause?

- ☐ 1 none
- ☐ 2
- ☐ 3
- ☐ 4
- ☐ 5 very much

If you have children:

How much has the COVID-19 pandemic changed your <u>relationship with children</u>?

- ☐ 1 not at all
- ☐ 2
- ☐ 3
- ☐ 4
- ☐ 5 very strongly

How stressful is this for you?	☐ 1 not at all stressful<br>☐ 2<br>☐ 3<br>☐ 4<br>☐ 5 very stressful
How much positive effect does this cause?	☐ 1 none<br>☐ 2<br>☐ 3<br>☐ 4<br>☐ 5 very much
**Social contacts:**	
How much has the COVID-19 pandemic changed your <u>social contacts</u>?	☐ 1 not at all<br>☐ 2<br>☐ 3<br>☐ 4<br>☐ 5 very strongly
How stressful is this for you?	☐ 1 not at all stressful<br>☐ 2<br>☐ 3<br>☐ 4<br>☐ 5 very stressful
How much positive effect does this cause?	☐ 1 none<br>☐ 2<br>☐ 3<br>☐ 4<br>☐ 5 very much
**Daily routine:**	
How much has the COVID-19 pandemic changed your <u>daily routines</u>?	☐ 1 not at all<br>☐ 2<br>☐ 3<br>☐ 4<br>☐ 5 very strongly
How stressful is this for you?	☐ 1 not at all stressful<br>☐ 2<br>☐ 3<br>☐ 4<br>☐ 5 very stressful

How much positive effect does this cause?	☐ 1 none<br>☐ 2<br>☐ 3<br>☐ 4<br>☐ 5 very much

Physical well-being:

How much has the COVID-19 pandemic changed your <u>physical well-being</u>?	☐ 1 not at all<br>☐ 2<br>☐ 3<br>☐ 4<br>☐ 5 very strongly
How stressful is this for you?	☐ 1 not at all stressful<br>☐ 2<br>☐ 3<br>☐ 4<br>☐ 5 very stressful
How much positive effect does this cause?	☐ 1 none<br>☐ 2<br>☐ 3<br>☐ 4<br>☐ 5 very much

Mental well-being:

How much has the COVID-19 pandemic changed your <u>mental well-being</u>?	☐ 1 not at all<br>☐ 2<br>☐ 3<br>☐ 4<br>☐ 5 very strongly
How stressful is this for you?	☐ 1 not at all stressful<br>☐ 2<br>☐ 3<br>☐ 4<br>☐ 5 very stressful
How much positive effect does this cause?	☐ 1 none<br>☐ 2<br>☐ 3<br>☐ 4<br>☐ 5 very much
